# Analysis of positive and negative allosteric modulation in metabotropic glutamate receptors 4 and 5 with a dual ligand

**DOI:** 10.1038/s41598-017-05095-5

**Published:** 2017-07-10

**Authors:** James A. R. Dalton, Jean-Philippe Pin, Jesús Giraldo

**Affiliations:** 1 Laboratory of Molecular Neuropharmacology and Bioinformatics, Institut de Neurociències and Unitat de Bioestadística, Universitat Autònoma de Barcelona, 08193 Bellaterra, Spain; 2 Network Biomedical Research Centre on Mental Health (CIBERSAM), Madrid, Spain; 3 Institute of Functional Genomics, Université de Montpellier, Unité Mixte de Recherche 5302 CNRS, Montpellier, France; 4 Unité de recherche U1191, INSERM, Montpellier, France

## Abstract

As class C GPCRs and regulators of synaptic activity, human metabotropic glutamate receptors (mGluRs) 4 and 5 are prime targets for allosteric modulation, with mGlu5 inhibition or mGlu4 stimulation potentially treating conditions like chronic pain and Parkinson’s disease. As an allosteric modulator that can bind both receptors, 2-Methyl-6-(phenylethynyl)pyridine (MPEP) is able to negatively modulate mGlu5 or positively modulate mGlu4. At a structural level, how it elicits these responses and how mGluRs undergo activation is unclear. Here, we employ homology modelling and 30 µs of atomistic molecular dynamics (MD) simulations to probe allosteric conformational change in mGlu4 and mGlu5, with and without docked MPEP. Our results identify several structural differences between mGlu4 and mGlu5, as well as key differences responsible for MPEP-mediated positive and negative allosteric modulation, respectively. A novel mechanism of mGlu4 activation is revealed, which may apply to all mGluRs in general. This involves conformational changes in TM3, TM4 and TM5, separation of intracellular loop 2 (ICL2) from ICL1/ICL3, and destabilization of the ionic-lock. On the other hand, mGlu5 experiences little disturbance when MPEP binds, maintaining its inactive state with reduced conformational fluctuation. In addition, when MPEP is absent, a lipid molecule can enter the mGlu5 allosteric pocket.

## Introduction

With eight subtypes, human metabotropic glutamate receptors (mGluRs) are involved in the modulation of pre- and postsynaptic neuronal activity through the binding of glutamate, the major excitatory neurotransmitter in the CNS^1, 2^. Part of the Class C G-protein coupled receptors (GPCRs) family, mGluRs form disulphide-linked homo-dimers^3^, where each protomer consists of an extracellular domain containing the orthosteric glutamate binding-site, a heptahelical transmembrane (TM) domain (analogous to Class A GPCRs)^4, 5^ with potential allosteric binding-site, and a cysteine-rich linking region in between^6^. Due to their involvement in neurological disorders such as schizophrenia and Alzheimer’s^7–9^, mGluRs represent attractive pharmacological targets for allosteric modulators or orthosteric ligands^10–12^. In particular, mGlu4 and mGlu5 are relevant targets for allosteric modulation because they functionally oppose each other, where mGlu4 negatively regulates adenylyl cyclase and mGlu5 positively regulates phospholipase C^13^. This means mGlu5 inhibition or mGlu4 stimulation can potentially treat anxiety^14–16^, chronic pain^17–21^ and Parkinson’s disease^22, 23^. Indeed, one of the most well characterized allosteric modulators that binds both receptors, 2-Methyl-6-(phenylethynyl)pyridine (MPEP), is a negative allosteric modulator (NAM) and inverse agonist of mGlu5 as well as a positive allosteric modulator (PAM) of mGlu4, giving it dual functional behaviour^24^. However, the way MPEP elicits these opposite allosteric effects is not well understood from a structural point of view, or indeed how other allosteric compounds can act as dual mGlu4 PAMs (or NAMs) and mGlu5 NAMs (or PAMs)^24–27^.

Recently, the crystal structures of inactive mGlu1 and mGlu5 TM domains have been determined at high resolution, with co-crystallized NAMs bound in their allosteric pockets^28–30^. This has allowed for the docking of MPEP into the TM domain of mGlu5^31^ and an mGlu4 TM homology model in order to probe possible binding modes^32, 33^. However, these modelling studies, as well as mGlu1 and mGlu5 crystal structures, reveal little about the activation process of mGluRs or specific mechanisms behind positive allosteric modulation. This is further complicated by mGluR activation occurring by two different means, either as homo-dimers *in vivo* ^6^, or single truncated TM domains *in vitro* ^5^. Our current understanding of mGluR homo-dimer activation proceeds first by the binding of glutamate (or agonist) to extracellular domains causing an inter-domain scissoring movement, signal transmission through cysteine-rich domains, and sequential inter-subunit and intra-subunit conformational changes of TM domains inside the membrane^6, 34, 35^. This process leads to receptor activation and G-protein binding, but at only one of the TM domains, not both^6^. On the contrary, truncated mGlu5 (as well as other truncated Class C GPCRs such as GABA-B2 or Ca(2+) receptors)^36, 37^ behaves in an identical fashion to Class A GPCRs, where its TM domain is directly activated by PAMs with subsequent G-protein recruitment, or directly inactivated by NAMs with concomitant decrease in basal activity^5^.

Courtesy of the size and complexity of mGluR homo-dimer structures, as well as the absence of a full homo-dimer crystal structure, investigating homo-dimer activation is very challenging. However, due to the smaller size of a truncated TM domain, and with recent mGlu1 and mGlu5 TM domain crystal structures, investigating activation and allosteric modulation of a TM domain is more achievable with current computational approaches^38–40^. As already demonstrated, long-timescale unbiased molecular dynamics (MD) simulations are a promising technique for determining ligand-dependent or allosteric conformational changes in GPCRs^38–45^. These highly adaptable proteins can adopt multiple conformational states e.g. several actives, intermediates and inactive states, either in response to the binding of different ligands or as part of their normal (apo) basal activity^39–41, 46, 47^. However, obtaining accurate MD simulations of GPCRs is dependent on several factors, such as sufficiently high sequence identity between target and template(s) for reliable homology modelling (ideally >40%)^48^, implementation of a suitable force-field for membrane proteins^49–51^, compatible ligand parameters^52–55^, and long simulation times to capture relevant receptor conformational changes. It has been suggested that a continuous trajectory in the microsecond range is necessary to capture meaningful conformational changes in GPCRs^38, 42, 44, 45^. Allosteric modulation in mGluRs, and Class C GPCRs in general, is of great interest from both mechanistic and pharmacological point-of-views, and is an area where such MD simulations can be useful.

**Figure 1:**
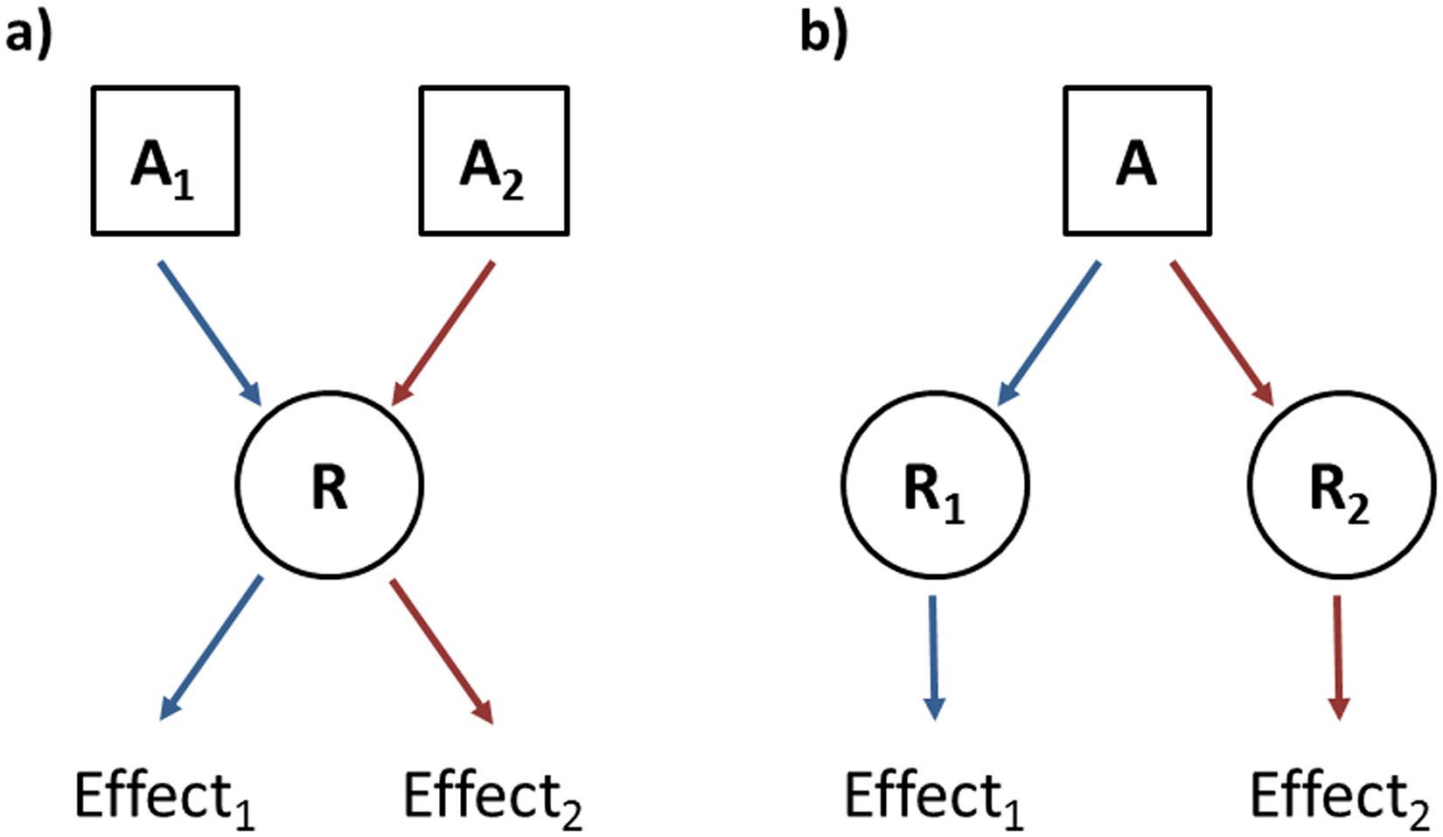
(**a**) Two agonists A1 and A2 activate the receptor R producing effects 1 and 2, respectively. (**b**) Agonist A activates receptors 1 and 2 producing effects 1 and 2, respectively. In the present study ligand A corresponds to MPEP whereas receptors 1 and 2 correspond to mGlu4 and mGlu5, respectively.

In light of recent successes of unbiased MD simulations capturing conformational changes in GPCRs^41, 43–45, 56^, including photoswitching of a light-sensitive NAM in mGlu5^31^, we employ long-timescale MD simulations for investigating allosteric modulation in the TM domains of mGlu4 and mGlu5. As the understanding of drug functionality in mGluRs is so difficult; two reasons being the shallow structure-activity relationships displayed by mGlu ligands and significant changes in efficacy mode upon minor changes in ligand molecular structure^57^, we follow a different strategy than the norm for analysing drug-receptor interactions. Instead of comparing the functionality of a collection of ligands in a single receptor, we analyse the functionality of a single ligand, MPEP, in two different receptors, mGlu4 and mGlu5. By doing so, we gain two benefits: first, we avoid the structure-activity noise that is present in ligand space associated with mGluRs and second, we focus on receptor recognition modes that may discriminate between PAM and NAM ligand functions. Thus, we change the perspective of the structure-activity analysis by providing structural variation at the receptor level rather than at the ligand level (Fig. 1). First, by homology modelling mGlu4 from the crystal structure of mGlu5^29^ and second, by performing a total of 30 µs of unbiased MD simulations, we identify several structural differences between mGlu4 and mGlu5, as well as key differences responsible for MPEP-mediated positive and negative allosteric modulation, respectively. In addition, a mechanism of mGlu4 TM domain receptor activation is suggested, which is perhaps applicable to all mGluRs and is supported by pre-existing experimental data^4, 5, 32, 35, 58–62^.

## Methods

### Homology modelling

The truncated mGlu5 TM domain crystal structure^29^ (PDB id: 4OO9) was converted into apo *wt* according to a previously described modelling protocol^31^, including completion of missing loops, removal of mavoglurant ligand, inclusion of co-crystallized water, and mutation of non-native sidechains. The apo mGlu4 TM domain was homology modelled from *wt* apo mGlu5 using ROSETTA v3.4^63^ with fragments generated with the ROBETTA webserver^64^, following an mGlu4-mGlu5 TM domain sequence alignment generated by PROMALS-3D^65^, manually curated for improved alignment accuracy (see SI).

### Docking

Coordinates for MPEP were generated with Maestro^66^. Autodock v4.2^67^ was used to dock MPEP into the apo mGlu4 and mGlu5 TM domain models, with flag “-U nphs_lps_nonstdres” to maintain co-crystallized waters. Grid points (40 × 40 × 90) were extended to cover total allosteric pocket volumes. The final docking conformation of MPEP in each receptor represents the top hit identified by best predicted affinity (nM) in the largest docking cluster. Subsequent energy minimization of docked structures was performed with CHIMERA^68^ in the AMBER-12SB force-field^69^.

### Molecular dynamics system setup

Four different receptor systems were generated using the CHARMM-GUI web-based interface^70^, each in a POPC membrane and solvated with TIP3P water molecules: apo mGlu4, apo mGlu5, mGlu4 with docked MPEP, mGlu5 with docked MPEP. Receptor structures were orientated according to the OPM database^71^ entry: 4oo9. Charge neutralizing ions (0.15 M KCl) were introduced to each system. Parameters of membrane, water and protein were automatically generated by CHARMM-GUI^70^ according to CHARMM36 force-field^49^ with ligand parameters automatically generated according to CHARMM36 General Force Field^52–54^. Ligand parameters were checked to comply with recommended accuracy thresholds^54, 55^.

### Molecular dynamics and analysis

Molecular dynamics (MD) simulations of mGlu4 and mGlu5, each embedded in a POPC membrane, with and without bound MPEP (representing four MD systems in total), were performed using the CHARMM36 force-field^49^ with ACEMD^72^ on specialized GPU-computer hardware. Each system was equilibrated for 28 ns at 300 K and 1 atm, with positional harmonic restraints on protein and ligand heavy atoms progressively released over the first 8 ns of equilibration and then continued without constraints. After equilibration, each system was subjected to an unbiased continuous production run of 5 µs under the same conditions. As an additional control, mGlu4 MD simulations were repeated to confirm observations (with and without bound MPEP). Simulation trajectories were analysed using VMD software v1.9.2^73^ to obtain root mean square deviation (RMSD) plots and root mean square fluctuation (RMSF) heatmaps. The following VMD plugins were used: “Hydrogen Bonds” to analyse protein-protein and protein-ligand H-bond occupancies (applied criteria of donor-acceptor distance ≤3.5 Å and 60° angle cut-off), “Timeline” to analyse secondary structure stability, “Collective variable analysis (Plumed)” for analysing inter-residue distances i.e. receptor ionic-locks, protein-ligand interactions, and protein-protein salt-bridges and H-bonds. The software *Helix Packing Pair* ^74, 75^ was used to calculate inter-helical angles between packed helix pairs i.e. helices that contain at least one inter-helical H-bond/vdW contact. Helices were defined by DSSP^76^.

### Calculation of average receptor conformation

Average protein conformations obtained during respective MD simulations of the four different systems (apo mGlu4, apo mGlu5, mGlu4-MPEP, mGlu5-MPEP) were generated with the TCL Trajectory Smooth 1.1 script (download source: www.ks.uiuc.edu/Research/vmd/script_library/scripts/trajectory_smooth/) executed within VMD v1.9.2^73^. The first half of each respective simulation was not considered in the calculation of the average conformation in order to allow each receptor protein to obtain its preferred conformational state. An averaging window was applied across the last 2.5 µs of each MD simulation to calculate average protein/ligand coordinates.

## Results

### Comparison of apo mGlu4 and mGlu5 structures and dynamics

First, missing loops in the mGlu5 TM domain (hereafter referred to as mGlu5) crystal structure were added as described in Methods, mutant residues reverted back to *wt*, and co-crystallized mavoglurant removed from the allosteric binding-site, generating an apo receptor state. The TM domain of apo mGlu4 (hereafter referred to as mGlu4) was homology modelled from the mGlu5 crystal structure with ROSETTA^77^ (see Methods). The sequence identity between mGlu4 and mGlu5 in their TM domains is 47% (SI Fig. 1), which indicates the mGlu4 homology model is sufficiently accurate^48^. The apo states of mGlu4 and mGlu5 were subjected to 5 µs MD simulations (twice in the case of mGlu4) in order to observe (inactive) receptor behaviour.

**Figure 2:**
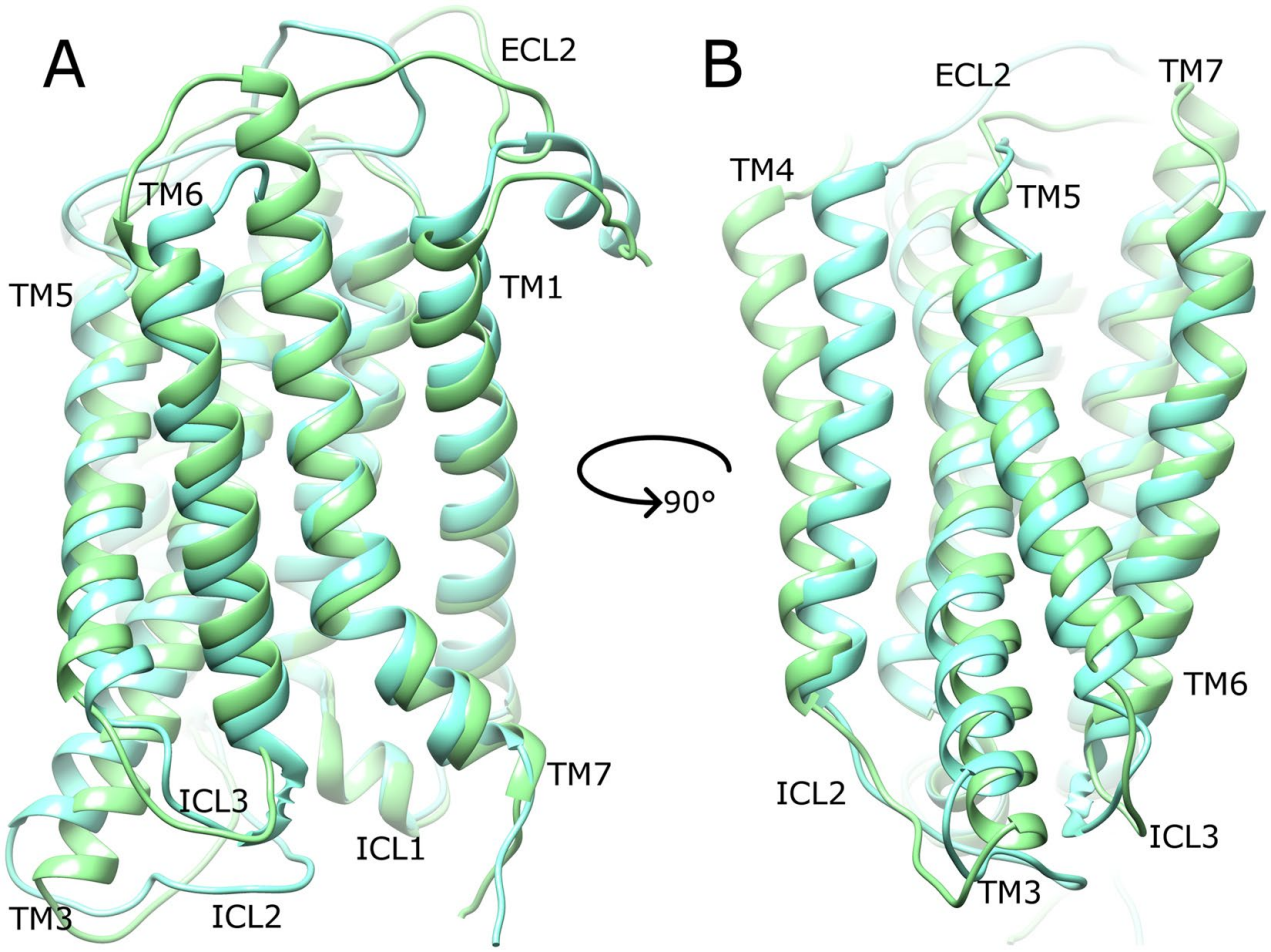
Average conformation of mGlu4 (light green) and mGlu5 (cyan) in their apo states, obtained from respective MD simulations, showing a 90° rotation between (**A**) and (**B**) around the membrane plane (extracellular-side: top, intracellular-side: bottom). Average receptor states are calculated from respective MD simulations (between 2.5–5 µs). Relevant structural features are labelled: extracellular loops (ECLs), intracellular loops (ICLs) and transmembrane helices (TMs).

After calculating receptor average states from the second half of their respective MD simulations (see Methods), mGlu4 and mGlu5 adopt similar conformations in their apo states (Fig. 2) albeit with some interesting differences. Firstly, mGlu4 has a longer extracellular loop 2 (ECL2) than mGlu5, whilst mGlu5 has a longer intracellular loop 2 (ICL2) than mGlu4. On the contrary, intracellular loops 1 and 3 (ICL1 and ICL3) are very similar in length and structure, with ICL1 predominantly helical in both. Other differences include mGlu4 having a longer TM7 (extending further on extracellular side) and a longer TM3 (extending further on intracellular side) than mGlu5. Other TM helices are observed to be similar in length between the two receptors. Interestingly, all TM helices adopt similar positions and orientations in both receptors, except for TM4, which in mGlu4 adopts a position partially more displaced from the rest of the helical bundle. Indeed, during its apo MD simulations, mGlu4 shows greater flexibility than mGlu5, both in terms of RMSD (~3 Å compared to ~1 Å, SI Fig. 2) and conformational fluctuation (RMSF, SI Fig. 3). In part, these effects could perhaps be explained by mGlu4 being a homology model while mGlu5 is a crystal structure. However, this does not satisfactorily explain the higher fluctuation of mGlu4, which is reproducibly observed from 1–5 µs (SI Figs 2 and 3). This is because unrepresentative conformations of GPCR homology models are expected to resolve within the first few hundred nanoseconds of MD simulations^78^. Furthermore, an analysis of mGlu4 secondary structure over time compares favourably with that of mGlu5 and reveals no structural instability (SI Fig. 4). Instead, it suggests the mGlu4 homology model is reliable and the higher flexibility observed in mGlu4 is a pertinent feature. In particular, a high degree of flexibility is observed in TM3 and TM4 of mGlu4, especially at their extracellular and intracellular ends where they go on to form loops ECL2 and ICL2, respectively. Although some conformational fluctuation is also observed in TM3 and TM4 of apo mGlu5, it is generally restricted to just the intracellular side (SI Fig. 3).

**Figure 3:**
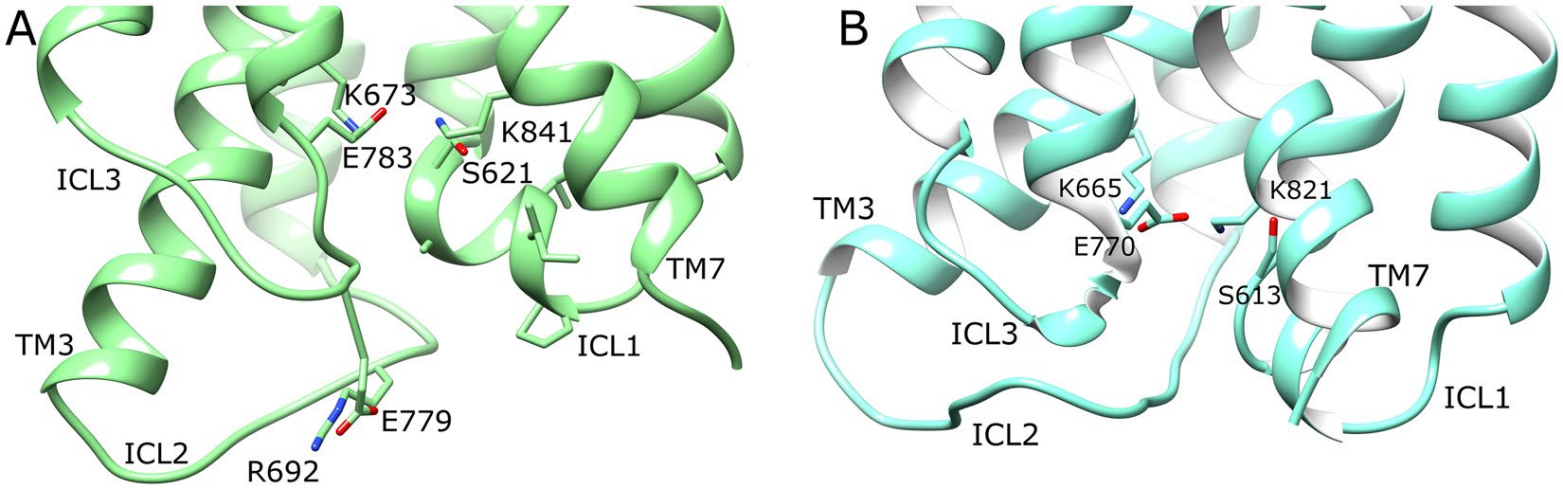
Average conformations of intracellular loops and selected charged/polar residues of (**A**) apo mGlu4 (green) and (**B**) apo mGlu5 (cyan), obtained from 2.5–5 µs of respective MD simulations. Relevant structural features are labelled, i.e. intracellular loops (ICLs) and intramolecular locks, i.e. K673-E783 in mGlu4 ionic-lock and K665-E770 in mGlu5 ionic-lock.

Regarding notable intramolecular interactions, mGlu4 contains an ionic-lock between residues K673 and E783 (K3.50 and E6.35; Pin GPCR class C numbering)^2, 79^ on helices 3 and 6, respectively. This is analogous to the ionic-lock of Class A GPCRs, which is thought to participate in G-protein binding when broken and stabilizes the inactive state when formed^4^. The mGluR ionic-lock was first confirmed in the crystal structure of mGlu1^30^ and later in the mGlu5 crystal structure^29^ (composed of residues K665/3.50 and E770/6.35), although its functional importance was initially characterized in the homologous GABA_B_ receptor^4^. MD simulations of both apo mGlu4 and mGlu5 confirm this ionic-lock to be a common stabilizing feature of the inactive state (Fig. 3), albeit with some minor fluctuation (SI Fig. 5). In addition to the ionic-lock, and unlike in mGlu5, mGlu4 possesses an extra inter-loop ionic interaction between ICL2 and ICL3, involving residues R692 and E779. This salt bridge remains stable for >3 microseconds in two different MD simulations of apo mGlu4 (SI Fig. 6). This interaction maintains ICL2 and ICL3 in close association, each with an inward conformation, restricting access to the intracellular side of the TM domain. This contributes to the inactivity of the apo state by hiding the ionic-lock and presumably prevents G-protein binding. Another common intracellular “locking” interaction observed in mGlu4 and mGlu5 occurs between ICL1 and TM7. In mGlu4, this polar interaction involves residues S621 and K841/7.51 and in mGlu5, S613 and K821/7.51 (Fig. 3). Over the course of respective MD simulations, an occupancy analysis of this H-bond (NH–O) reveals it to be more stable in mGlu5 (63%) than mGlu4 (27%). Between this and the adjacent ionic-lock, the four participating structural elements: TM3, TM6, TM7 and ICL1 are locked together in a tight conformation that persists throughout the course of all MD simulations of apo mGlu4 and mGlu5. In the same way that mutations in the ionic-lock of mGlu5 (either K665/3.50 or E770/6.35) generate increased basal receptor activity^4^, mutations to S613 also do the same^29^, presumably via disruption of this four-way interaction network and destabilization of the inactive state. In summary, apo mGlu5 and mGlu4 can be characterized in general terms by tightly associated TMs and ICLs and two or more intramolecular “locking” interactions, which help stabilize the inactive state.

### Comparison of MPEP binding in mGlu4 and mGlu5

**Figure 4:**
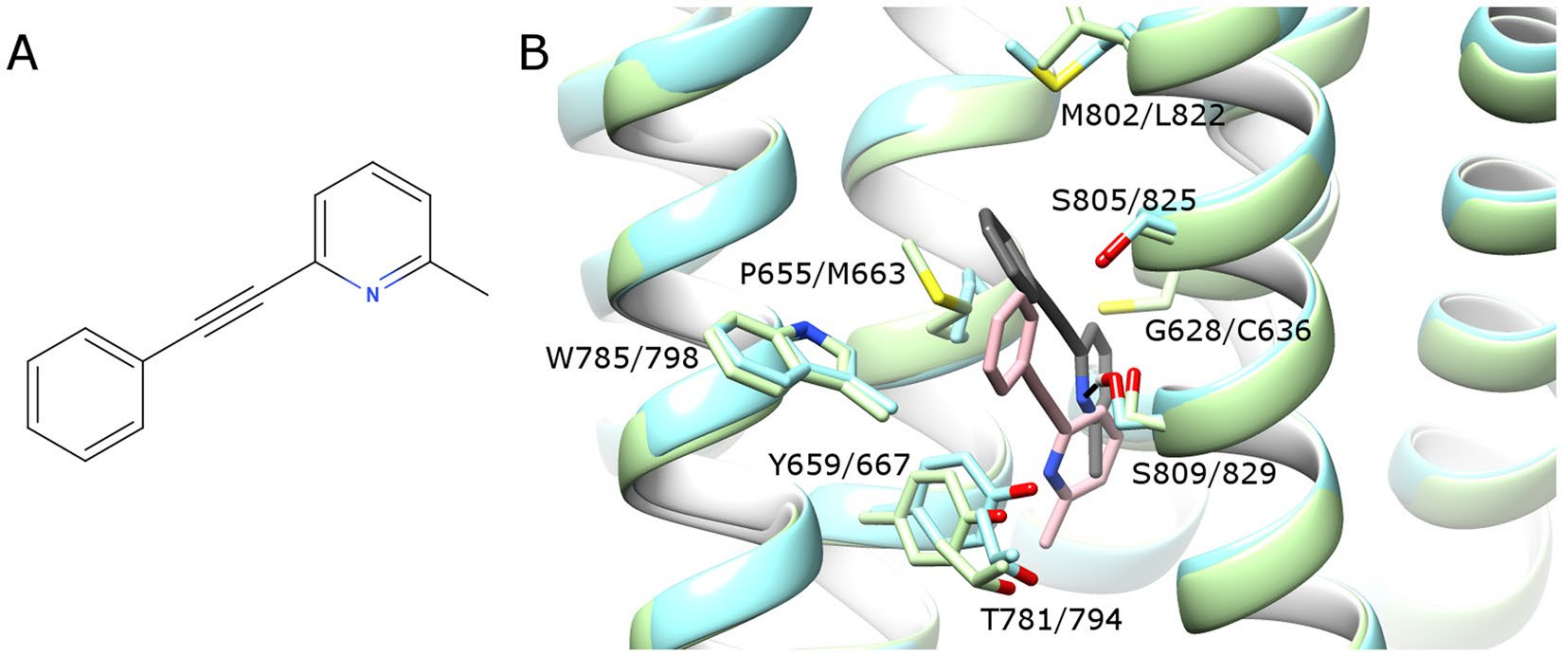
(**A**) 2-Methyl-6-(phenylethynyl)pyridine (MPEP), (**B**) comparison of MPEP (grey) docking in mGlu5 (light blue), and MPEP (pink) docking in mGlu4 (light green) prior to MD simulations. Selected residues delineating allosteric pockets or participating in ligand binding are labelled (labelling: mGlu5 first, mGlu4 second). Protein-ligand H-bonds are represented by black lines. Helix orientations as follows: TM5 left, TM3 centre-left, TM7 centre-right, TM1 right. TM6 backbone is hidden.

Docking of MPEP into mGlu5 was performed as reported previously^31^ with Autodock4.2^67^, yielding a docking pose at the bottom of the allosteric pocket, consistent with NAM-bound mGlu5 crystal structures^28–30, 33^. Using the same methodology, MPEP was docked into the allosteric pocket of mGlu4, generating a docking pose at the bottom that is consistent with previously published experimental information^32^ and in agreement with a previous docking of MPEP in a different mGlu4 homology model^32^. As mGlu4 was homology modelled from a NAM-bound crystal structure of mGlu5, it is likely that this homology model also resembles an inactive receptor state and is not optimal for docking mGlu4 PAMs. It is therefore logical that the docking score of MPEP in mGlu4 is not quite as favourable as in mGlu5 (predicted difference of ~0.4 kcal/mol, SI Table 1) despite the two docking poses being similar (Fig. 4).

**Figure 5:**
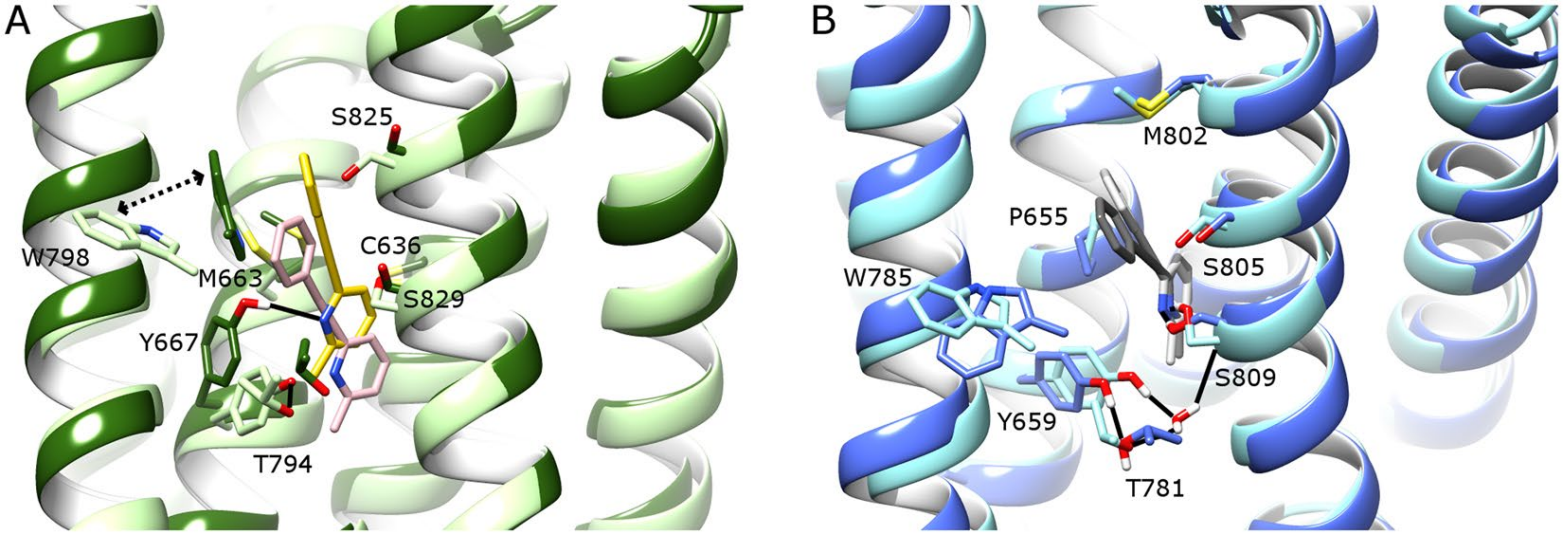
Comparison of bound MPEP in (**A**) mGlu4 and (**B**) mGlu5, before and after respective MD simulations (average conformations calculated from 2.5–5 µs). Colours as follows: (**A**) MPEP before MD in pink, MPEP after MD in yellow, mGlu4 before MD in light green, mGlu4 after MD in dark green; (**B**) MPEP before MD in dark grey, MPEP after MD in light grey, mGlu5 before MD in light blue, mGlu5 after MD in blue. Selected residues delineating allosteric pockets or participating in ligand binding are labelled. Protein-ligand H-bonds are represented by solid black lines. The inward movement of W798 in mGlu4 is indicated with a dotted arrow. Helix orientations as follows: TM5 left, TM3 centre-left, TM7 centre-right, TM1 right. TM6 backbone is hidden.

During a 5 µs MD simulation, MPEP remains highly stable in mGlu5, remaining very close to the original docking position with an RMSD of 0.5–2.0 Å (SI Fig. 6), making a protein-ligand H-bond (89% occupancy) with S809/7.39 on TM7 via its pyridine ring (Fig. 5 and SI Fig. 7). This confirms the same observation made previously^31^, however replicated here over a longer time period. The original crystal structure of mGlu5 contains a water-mediated H-bond between TM3 (Y659/3.44) and TM6 (T781/6.46) in the core of the receptor, which stabilizes the inactive state^29^. Over the course of the MPEP-bound mGlu5 simulation, this co-crystallized water molecule is lost from the allosteric binding pocket, and instead a direct H-bond with 39% occupancy is formed between the same two residues (Fig. 5 and SI Fig. 8). This appears to preserve the functionality of the observed crystal structure interaction (maintaining TM3-TM6 distance) and indicates that MPEP behaves in a similar fashion in mGlu5 as the co-crystallized NAM mavoglurant^29^. Likewise, the rest of the mGlu5 allosteric pocket remains relatively unchanged with respect to the original crystal structure, including an outward positioning of W785/6.50 (Fig. 5), which is mostly observed after 2 µs (SI Fig. 7).

As mGlu4 was homology modelled from the NAM-bound crystal structure of mGlu5^29^, and MPEP is a PAM in mGlu4, it was expected that during MD simulations of MPEP-bound mGlu4, the fit between ligand and receptor might change to reflect different ligand function. Indeed, this occurs as MPEP changes its orientation within the allosteric binding pocket, moving ~2.5 Å higher from its initial docking position in the first few nanoseconds (Fig. 5), making an H-bond with Y667/3.44 on TM3 (49% occupancy, SI Fig. 7). Over the course of MD simulations, the ligand displays an average RMSD of 2.2 Å with a range of 1.5–3.5 Å in mGlu4 (SI Fig. 2). The initial movement of the ligand has the effect of also changing the conformation of the mGlu4 allosteric pocket, where W798/6.50 moves inwards to make a ring-stacking interaction with MPEP (Fig. 5 and SI Fig. 7), and the originally modelled H-bond between Y667/3.44 on TM3 and T794/6.46 on TM6 (analogous to the Y659/3.44-T781/6.46 interaction in MPEP-bound mGlu5) is broken. This is because of the newly formed H-bond between MPEP and Y667/3.44 (Fig. 5 and SI Fig. 7). In contrast in mGlu5, the observed protein-ligand H-bond with S809/7.39 does not disturb the adjacent TM3-TM6 inter-helical H-bond (SI Fig. 8).

**Figure 6:**
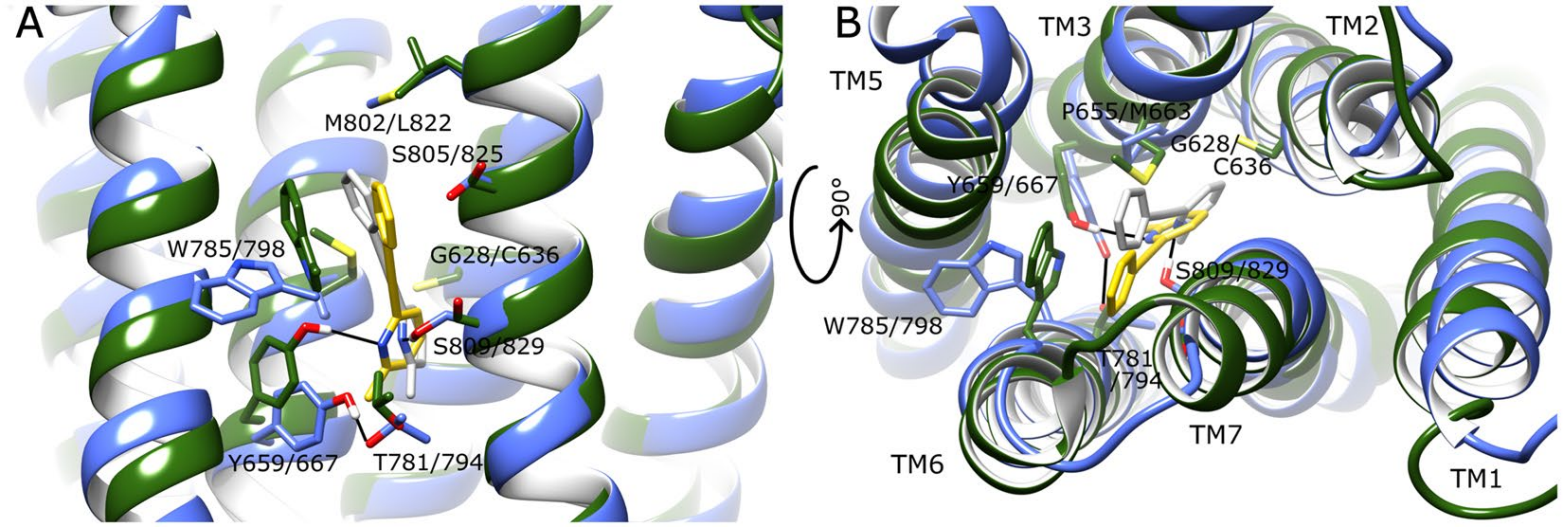
A comparison of average conformations of mGlu4 with bound MPEP (green, yellow, respectively) and mGlu5 with bound MPEP (blue, grey, respectively) from 2.5–5 µs of respective MD simulations from following perspectives: (**A**) side-view within membrane and (**B**) extracellular top-view. Selected residues delineating allosteric pockets or participating in ligand binding are labelled (mGlu5 first, mGlu4 second). Protein-ligand H-bonds are represented by black lines.

When the binding modes of MPEP in mGlu4 and mGlu5 are directly compared from respective 5 µs MD simulations, the ligand is seen to occupy the same space between TM3 and TM7 at the bottom of both allosteric pockets with pyridine rings partially overlapping (Fig. 6). However in mGlu4, the phenyl ring of MPEP adopts a position 3.0 Å closer to TM6 and its methyl group 1.5 Å more distant from TM2. The residue most responsible for this difference in mGlu4 appears to be C636/2.49 on TM2, which reduces the space at the bottom of the allosteric pocket compared to mGlu5 where the equivalent residue is G628/2.49 (Fig. 6). This has the effect of pushing MPEP in mGlu4 closer to Y667/3.44 where it can make a protein-ligand H-bond, whereas in mGlu5 the equivalent residue (Y659/3.44) instead makes an inter-helical H-bond with T781/6.46 (Fig. 6 and SI Fig. 8). The protein-ligand H-bond between MPEP and S809/7.39 in mGlu5 is not observed in MPEP-bound mGlu4, where the equivalent residue S829/7.39 points away from the allosteric pocket (Fig. 6).

### Comparison of dynamics between MPEP-bound and apo states of mGlu4 and mGlu5

**Figure 7:**
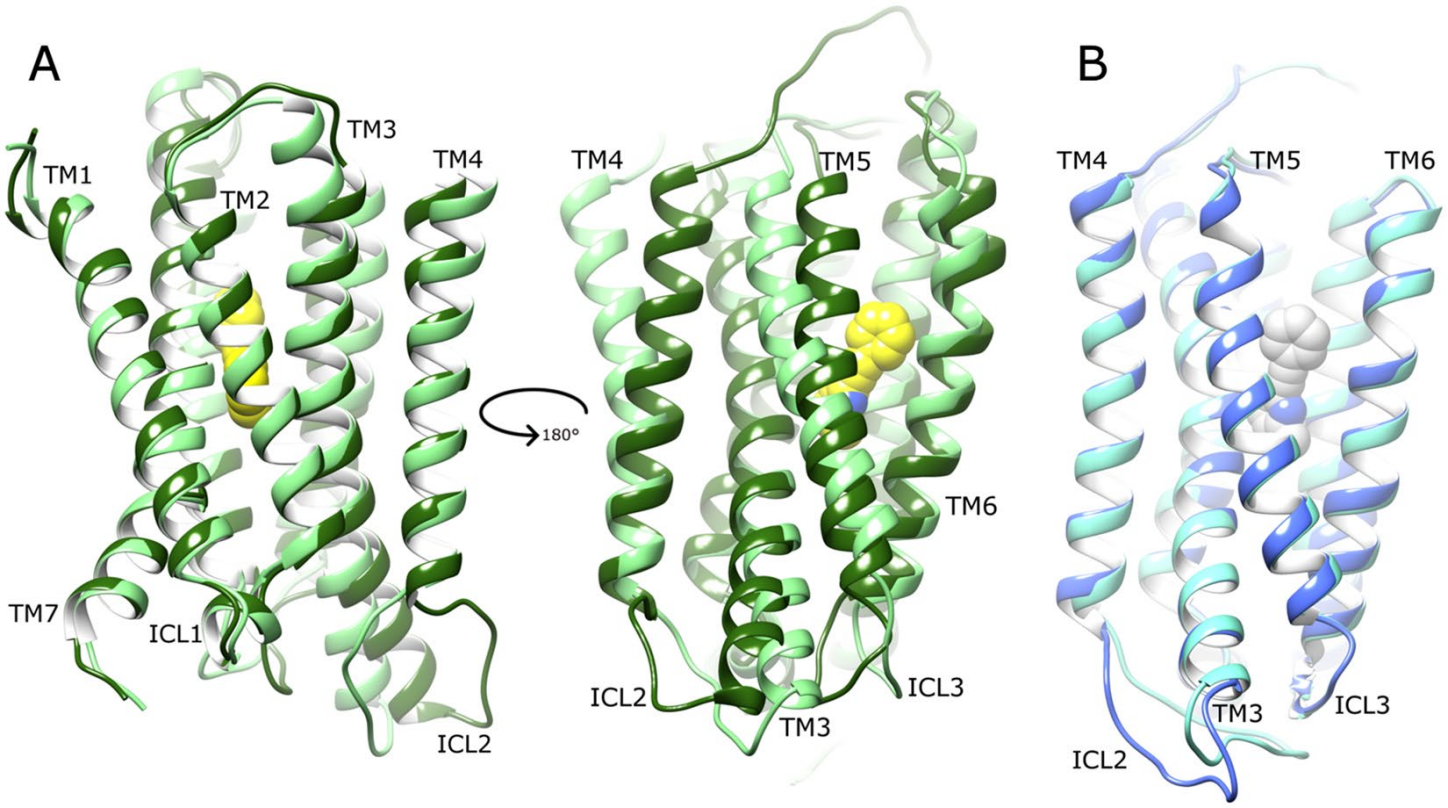
Comparison of average conformations of (**A**) mGlu4 with bound MPEP (dark green, yellow, respectively) and apo mGlu4 without MPEP (light green) and (**B**) mGlu5 with bound MPEP (blue, light grey, respectively) and apo mGlu5 without MPEP (cyan), obtained from 2.5–5 µs of respective MD simulations. A 180° rotation around the membrane plane of mGlu4 allows differences to be noted from opposite angles. Receptors are viewed from side, within membrane, with extracellular-side, top, and intracellular-side, bottom. Relevant structural features are labelled, i.e. intracellular loops (ICLs) and transmembrane helices (TMs).

**Figure 8:**
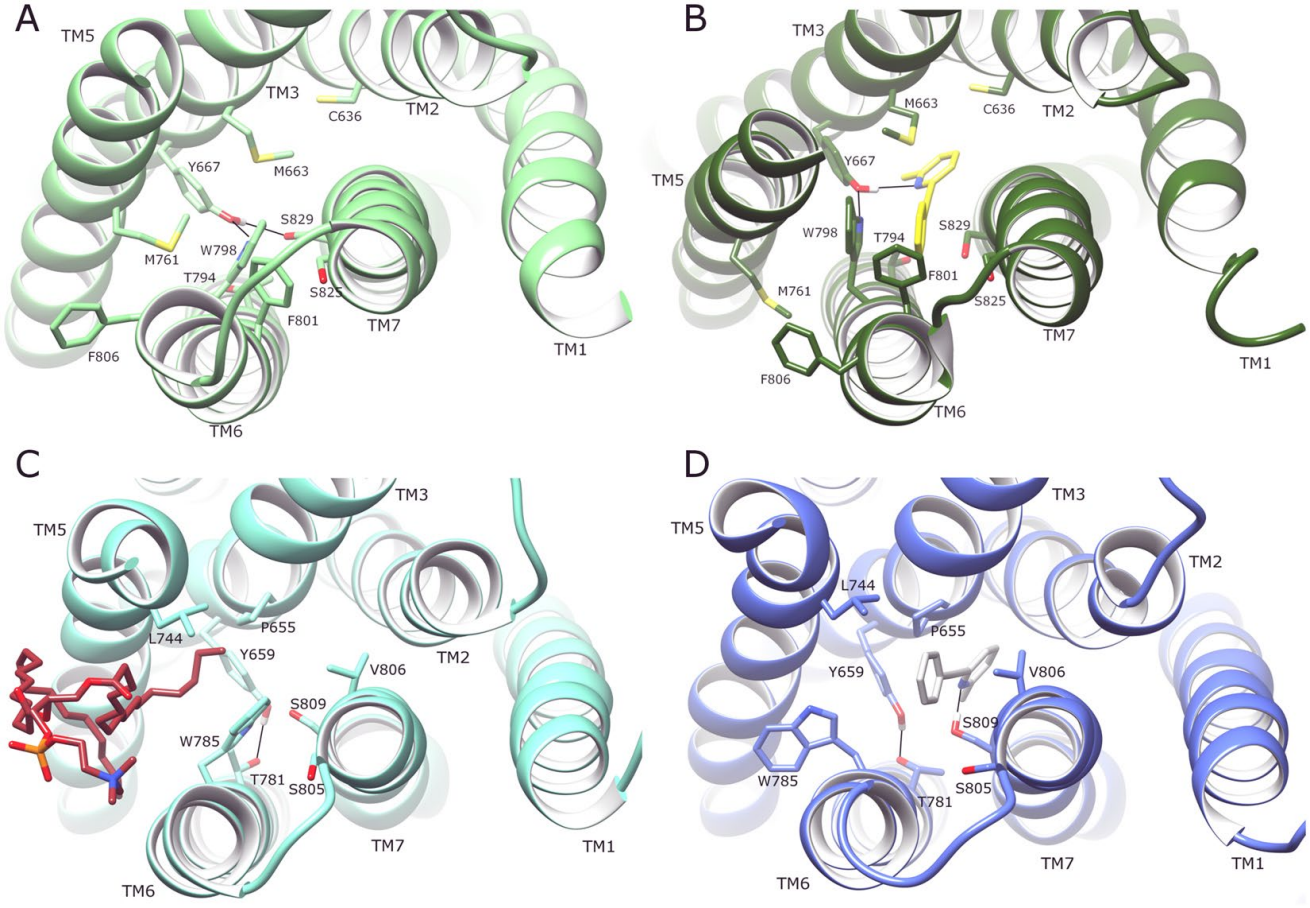
Comparison of average conformations of (**A**) apo mGlu4 without MPEP (light green), (**B**) mGlu4 with bound MPEP (dark green, yellow, respectively), (**C**) apo mGlu5 without MPEP (cyan) with bound lipid molecule (brown), (**D**) mGlu5 with bound MPEP (blue, light grey, respectively), each obtained from 2.5–5 µs of respective MD simulations. Receptors are viewed extracellular-side from top. Relevant structural features are labelled, i.e. transmembrane helices (TMs) and residues in the allosteric pocket interacting with lipid/MPEP.

In order to understand the allosteric effects of MPEP in both mGlu4 and mGlu5, a comparison between average MPEP-bound and apo states of each receptor can be made from their respective MD simulations (Fig. 7). In addition, receptor conformational fluctuation can be measured in terms of RMSF, and changes in protein secondary structure and inter-helical angles can be calculated. Together, these can give an indication of receptor conformational stability in ligand bound and apo states over time. Considering mGlu4 first, its average conformation with bound MPEP shows that the ligand increases the gap between TM3 and TM7 by ~2 Å with respect to the apo state (11.6 Å compared to 9.7 Å, as measured between Cα atoms of Y667/3.44 and S829/7.39 in MPEP-bound and apo states, respectively). This also causes a partial disordering of TM3 by one helical turn at its extracellular end (Fig. 7). This separation of TM3 and TM7 prevents the formation of an inter-helical H-bond at the bottom of the allosteric pocket, which is observed in the apo state between residues Y667/3.44 and S829/7.39 and changes its H-bond occupancy from 36% to 0% (Fig. 8 and SI Fig. 8). At the same time, the phenyl ring of MPEP packs against TM6 making contact with residues W798/6.50 and F801/6.53 in particular. In doing so, TM6 rotates towards TM5, altering the packing between these two helices and moving residues such as M761/5.48 and F806/6.58 into new positions (Fig. 8). This rotational movement can be seen as analogous to the rotation of TM6 observed in the activation of some Class A GPCRs^80^. However, the concomitant bending of TM6 in Class A GPCRs^81, 82^ is not observed here. In contrast, TM6 of mGlu4 is comparatively rigid.

The local effects of MPEP in the rearrangement of the mGlu4 allosteric pocket are seen to propagate through the rest of the receptor, affecting the orientations of TM3, TM4 and TM5. In particular, TM4 tilts by ~15° towards TM3 (SI Fig. 9). Likewise, TM5 tilts by ~12° towards TM6 (SI Fig. 9) while translating ~3 Å to the intracellular side (Fig. 7). In this new arrangement, the intracellular ends of TM5 and TM6 become ~2 Å closer (measured between Cα atoms of A781 and A784, see SI Figs 9 and 10). Taken together, these helical rearrangements, particularly the movements of TM4 and TM5, create a tighter receptor conformation (Fig. 7). Interestingly, TM1, TM2 and TM7 do not show significant conformational change in the MPEP-bound state of mGlu4, as is typically observed in Class A GPCRs when an agonist binds^75^. In particular, these helical rearrangements in mGlu4 affect the behaviour of ICL2 (the longest intracellular loop), which is seen to adopt a more outward conformation relative to the apo state (Fig. 7). This loop movement is primarily mediated by TM3 bending and TM4 tilting, which brings their intracellular ends ~3 Å closer together (measured between Cα atoms of R676 and P696, see SI Fig. 9). The ionic-lock of mGlu4 is also disrupted, particularly in the first half of its respective simulations (SI Fig. 5), with an average N–O distance of 3.9 Å when MPEP is bound compared to 3.3 Å in the apo state (respective ranges: 2.5–9.2 Å and 2.5–5.9 Å). This represents a notable change in the mGlu4 conformational ensemble and suggests positive modulation of receptor state. The concomitant conformational change in ICL2 is also potentially important as this loop is known to be involved in the activation of mGluRs and recognition of G-proteins^35, 59, 61^.

In contrast to mGlu4, mGlu5 experiences remarkably little disturbance in overall conformation whether MPEP is bound or not (Fig. 7). This suggests MPEP does not alter the apo conformation of mGlu5, rather just stabilizes the inactive state with reduced receptor conformational fluctuation that may reflect its inverse agonist activity (SI Fig. 3). An analysis of the mGlu5 ionic-lock reveals it to be similarly stable in both apo and MPEP-bound states (SI Fig. 5) with an average N–O distance of 3.3 Å in each case (respective ranges: 2.5–6.5 Å and 2.5–5.6 Å). Regarding other intramolecular interactions, an inter-helical H-bond is formed between TM3 (Y659/3.44) and TM6 (T781/6.46) at the bottom of the allosteric pocket in both states (Fig. 8 and SI Fig. 8). This interaction is the same as observed in the crystal structures of mGlu5 bound to NAMs mavoglurant and HTL14242, although mediated via a co-crystallized water molecule^28, 29^. During our MD simulations, this water molecule is observed transiently moving in and out of its crystallized position but eventually leaves the allosteric pocket. As previously mentioned, MPEP is seen to make a stable protein-ligand H-bond with TM7 (S809/7.39) (as other co-crystallized mGlu5 NAMs)^28, 29^ but this does not disrupt the inter-helical TM3-TM6 H-bond, which means the conformation of the allosteric pocket is much alike in both apo and MPEP-bound states (Fig. 8). Consequently, almost no change is observed in the receptor as a whole when MPEP is bound (Fig. 7) and suggests stabilization of the receptor in an inactive state. Interestingly, in the mGlu5 apo state, which experiences higher receptor conformational fluctuation than its MPEP-bound state (SI Fig. 3), there is enough flexibility for W785/6.50 to swing inwards inside the allosteric pocket from its initial outward position. This movement creates a gap between TM5 and TM6 that W785/6.50 previously filled and allows a lipid molecule to partially enter the allosteric pocket. This occurs through six terminal carbons of one lipid fatty-acid chain intruding ~5.5 Å into the allosteric pocket through the space vacated by W785/6.50 and between flanking residues G745/5.45, G748/5.48 on TM5, and L786/6.51, V789/6.54 on TM6. Inside the allosteric pocket, the fatty-acid chain is able to make direct contacts with residues L744/5.44, N747/5.47 (on TM5), P655/3.40 (on TM3), and W785/6.50 (on TM6). The other ten carbons of the same fatty-acid chain bind in an external surface cleft formed between TM5 and TM6. These protein-lipid interactions are observed to form after 2 µs and continue throughout the rest of the simulation. The potential function of these interactions is unknown but may offer allosteric influences over the receptor in its apo state. Lipid binding is not observed in the MPEP-bound mGlu5 state, where the ligand reduces receptor conformational flexibility (SI Fig. 3) and increases the stability of W785/6.50 in its outward position (Fig. 8 and SI Fig. 7).

### Comparison of dynamics of MPEP-bound mGlu4 and mGlu5

**Figure 9:**
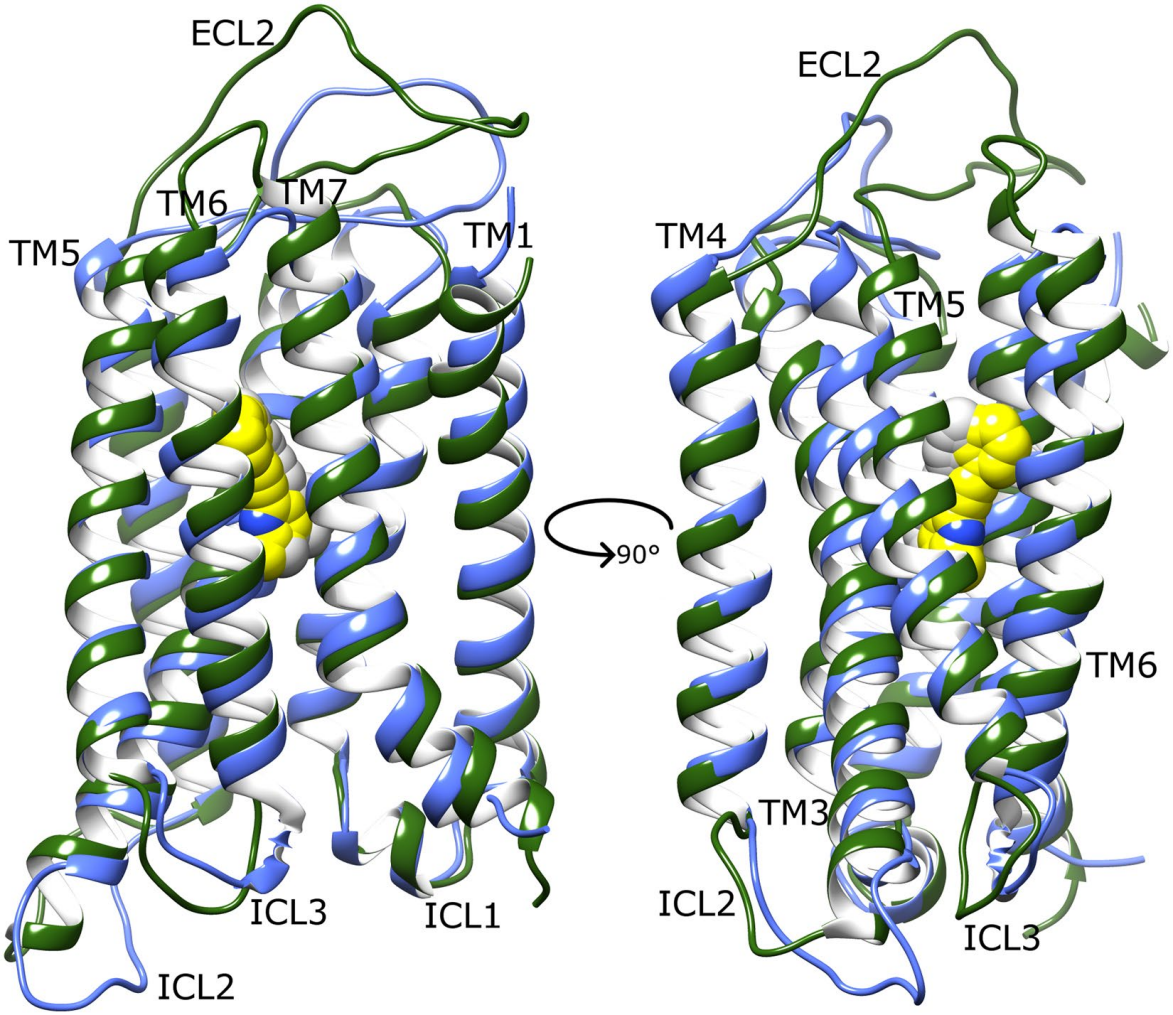
Comparison of average conformations of (**A**) mGlu4 with bound with MPEP (dark green, yellow, respectively) and mGlu5 bound with MPEP (blue, grey, respectively), obtained from 2.5–5 µs of respective MD simulations. Receptors are viewed from side, within membrane, with a 90° rotation around membrane plane for alternative views (left and right images). Relevant structural features are labelled, i.e. intracellular loops (ICLs) and transmembrane helices (TMs).

**Figure 10:**
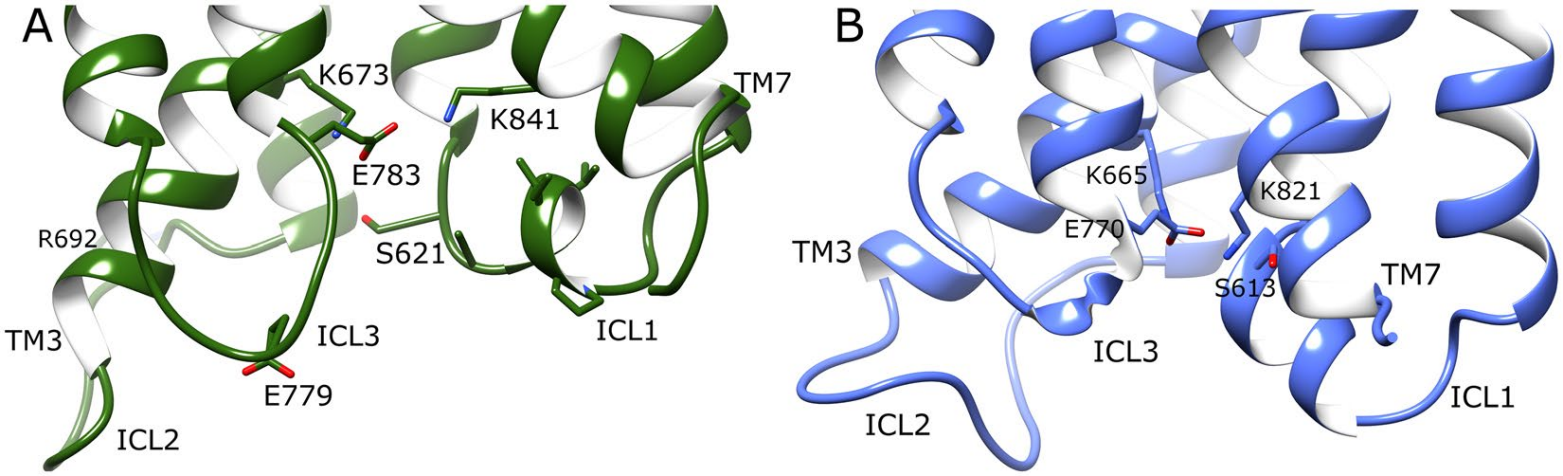
Average conformations of intracellular loops and selected charged/polar residues of (**A**) MPEP-bound mGlu4 (green) and (**B**) MPEP-bound mGlu5 (blue), obtained from 2.5–5 µs of respective MD simulations. Relevant structural features and residues are labelled, i.e. intracellular loops (ICLs), and ionic-locks: K673-E783 in mGlu4 and K665-E770 in mGlu5.

**Figure 11:**
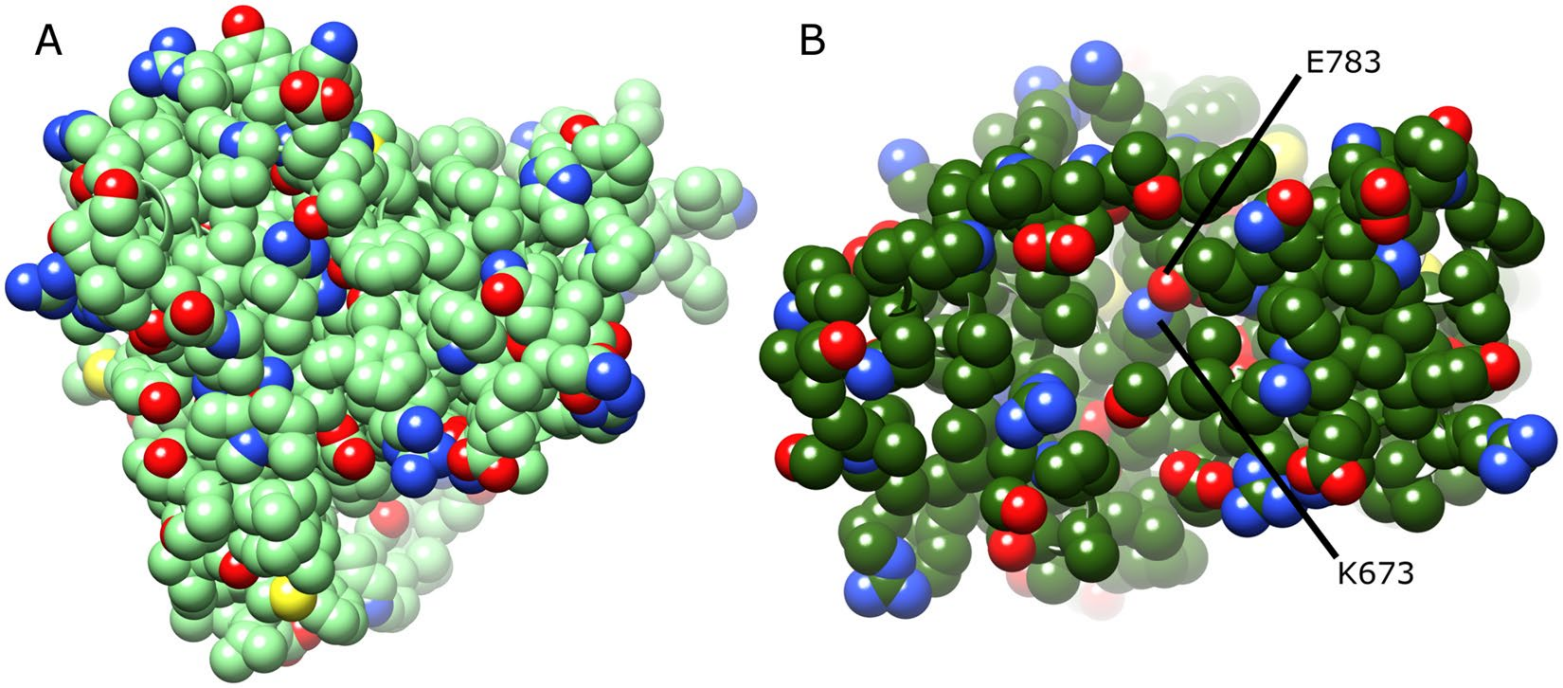
Comparison of average conformations of (**A**) apo mGlu4 without MPEP (green) and (**B**) mGlu4 with bound MPEP (dark green) obtained from 2.5–5 µs respective MD simulations. Receptors are viewed from their intracellular side with atoms displayed as spheres. Residues participating in the ionic-lock are labelled in (**B**).

When the average conformations of both MPEP-bound states of mGlu4 and mGlu5 are directly compared, the overall effect of positive allosteric modulation in mGlu4 can be seen in context of negative allosteric modulation in mGlu5 (Fig. 9). Interestingly, the conformational changes resulting from MPEP binding in mGlu4 make its allosteric pocket more tightly packed, similar to mGlu5, particularly regarding TM4. In addition, conformational bending of TM3 observed in MPEP-bound mGlu4 results in a close conformational match with TM3 of MPEP-bound mGlu5. Nevertheless, MPEP-bound mGlu4 and mGlu5 conformational states are not identical, as differences are observed in TM5, which is orientated more vertically in mGlu4, as well as TM6, which occupies a partially more outward position (Fig. 9). Most significantly, the largest differences are observed on the intracellular side, particularly ICL2, which has an outward conformation in MPEP-bound mGlu4, but a more inward conformation in MPEP-bound mGlu5 (Figs 9 and 10). As a result, the mGlu4 inter-loop ionic interaction between ICL2 (R692) and ICL3 (E779) is broken (Fig. 10 and SI Fig. 6). In addition, subtle but relevant conformational changes occur in ICL1 of MPEP-bound mGlu4 where loop helical structure is progressively lost (SI Fig. 4). This results in the disruption of the polar interaction between ICL1 (S621) and TM7 (K841/7.51) and reduces its H-bond (O–N) occupancy to just 5% (Fig. 10 and SI Fig. 6). In mGlu5, the equivalent polar interaction (S613 and K821/7.51) is unchanged when MPEP binds (Fig. 10 and SI Fig. 6) and has 54% H-bond occupancy. In addition, the helical structure of ICL1 in mGlu5 is maintained (SI Fig. 4). As previously mentioned, the ionic-lock in mGlu4 (between K673/3.50 and E783/6.35) is disrupted although not permanently broken (Fig. 10 and SI Fig. 5). On the other hand, in mGlu5, the ionic-lock (between K665/3.50 and E770/6.35) is unaffected (Fig. 10 and SI Fig. 5). The sum of conformational changes observed in mGlu4 creates a physical separation between ICLs and opening of the TM domain at its intracellular side (Fig. 11). This exposes the ionic-lock residues, K673/3.50 and E783/6.35. Furthermore, the same conformational changes are observed in two separate MD simulations of MPEP-bound mGlu4, indicating its reproducibility (SI Fig. 11). This new receptor conformation may facilitate easier binding of a G-protein α-subunit.

## Discussion

Metabotropic glutamate receptors are constitutive multi-domain homo-dimers *in vivo* but their monomeric truncated TM domains still function *in vitro*, responding to PAMs or NAMs as if they were traditional agonists or antagonists^5^. As such, we have performed a computational analysis, using long-timescale molecular dynamics (MD), to investigate positive and negative allosteric modulation of mGlu4 and mGlu5 TM domains. Furthermore, we have used the same allosteric modulator, MPEP, which acts positively in one receptor (mGlu4) and negatively in the other (mGlu5). To our knowledge, for the first time, we present a structural and mechanistic explanation as to why and how this is the case. As a result, we have identified reproducible conformational changes that occur during positive allosteric modulation of mGlu4, as part of receptor activation, with apo mGlu4 acting as a control. Unlike with Class A GPCRs, there is currently no crystal structure data for mGluRs in their active state (and class C GPCRs in general), so revealing features of mGluR activation is an important step in better understanding this family of receptors. It also allows for the identification of key similarities/differences between Class C and Class A GPCRs, such as ionic-locks^4^ and ICLs^59^, which have received attention in recent studies of Class A GPCRs^41, 42^.

Although both mGlu4 and mGlu5 belong to the same family and share 47% sequence identity in their TM domains, after homology modelling and MD simulations, they show several key differences, which include different lengths of ICLs, ECLs and TM helices. In particular, mGlu4 has longer TM3 and TM7 helices than mGlu5, longer ECL2, and shorter ICL2. Perhaps more importantly, compared to mGlu5, when no allosteric ligand is bound, mGlu4 appears to have a less compact TM domain and greater flexibility. These aspects may be linked at a functional level allowing these receptors to behave differently in terms of activation. The intracellular conformation of ICLs is important, as this is the area where G-proteins bind^4, 35, 59^. After MD simulations, both receptors in their apo state become stabilized in an inactive conformation. This inactive state can be characterized by an inward positioning of ICL2, helical ICL1, and two intramolecular locking interactions between TM3-TM6 (the ionic-lock^4^) and TM7-ICL1, respectively. Furthermore, an additional inter-loop ionic interaction between ICL2 and ICL3 is observed in mGlu4, which may provide additional control of mGlu4 activity. Taken together, these intracellular features result in the “closure” of the G-protein binding pocket in both receptors.

From our study, MPEP docks in mGlu4 and mGlu5 at the bottom of their allosteric pockets. However, despite similar docking poses, MPEP achieves a slightly better docking score in mGlu5 than in mGlu4. This is understandable as MPEP is a PAM in mGlu4 and a NAM in mGlu5. Therefore the conformational state of the receptor should have a bearing on how well the ligand docks. As the mGlu5 crystal structure is in an inactive state, it can be assumed that the homology model of mGlu4 is in an equivalent state, which might disfavour the docking of PAMs such as MPEP. In reality, docking into a single conformation of a receptor only yields limited information. However, through the use of long-timescale MD simulations, more information about the interplay between receptor and allosteric modulator can be revealed. In mGlu5, bound MPEP not only stabilizes the inactive state, but also restricts conformational fluctuation, thereby preventing access to other more active-like receptor states. This is in agreement with the inverse agonist activity of MPEP in mGlu5. This action is most probably facilitated by MPEP making an H-bond with TM7, as well as stable aromatic contacts with TM3 and TM6. This is in contrast to the apo state, which although remains in an inactive state overall, is more flexible with increased conformational fluctuation. The same observation can be made in mGlu4, albeit with different end states. Although its apo state remains inactive overall, the receptor displays more conformational fluctuation than mGlu5, particularly with regard to TM4. On the contrary, in mGlu4, MPEP induces/selects conformational change that results in a receptor conformation best described as “semi-active” (without bound G-protein, observation of a fully active GPCR conformation is unlikely)^38^. Once this conformation is obtained after approximately 2.5 µs, the receptor remains relatively stable with less fluctuation than the apo state and does not de-activate. Intriguingly, the binding of MPEP to mGlu4 creates a more tightly packed allosteric pocket with closer contacts between TM3, TM4 and TM5 as well as an H-bond between MPEP and TM3.

Despite some similarities in their TM domains, MPEP-bound mGlu4 and mGlu5 obtain different conformations in their intracellular loops and ionic-locks. In mGlu4, bound MPEP destabilizes ICL1 and stabilizes an outward position of ICL2, breaking two of three intramolecular locking interactions (between ICL2-ICL3 and ICL1-TM7) and destabilizing its third: the ionic-lock between TM3 and TM6. This receptor conformational ensemble is notably different to the mGlu4 apo state. In mGlu5, ICL1 and two intramolecular locks (between ICL1-TM7 and TM3-TM6) are undisturbed by MPEP binding, while ICL2 maintains an inward conformation, highly similar to the apo state. The MPEP-bound mGlu4 conformation appears to be partially active because separation and disordering of ICLs results in the widening of its intracellular binding pocket, exposing the ionic-lock residue LYS673/3.50. This receptor conformation is likely suited for binding G-proteins, which presumably require access to the ionic-lock in between ICLs, as observed in activated Class A GPCRs^75^. As the position of ICL2 appears to be significant in the process of mGlu4 activation^59^, the question of how MPEP regulates this outward loop movement arises. This appears to be through a sequence of events that begins with MPEP H-bonding to TM3, via the highly conserved residue Y667/3.44. This results in the tyrosine residue undergoing an upward and outward movement. This causes closer interaction between TM3 and TM4 at their intracellular ends, partly through a bending of TM3. Finally, this influences ICL2 to adopt an outward position. In Class A GPCRs, helical bending is often seen with TM6 during receptor activation, not TM3. This begs the question of whether TM3 in mGlu4 plays an analogous role in receptor activation, and maybe likewise in other Class C GPCRs. On the contrary, it is interesting to note that TM6 in both mGlu4 and mGlu5 is relatively short and rigid. This makes it unlikely that TM6 in mGlu4 and mGlu5 plays the same role as TM6 in Class A GPCRs^75^.

On a wider note, Y3.44 appears to be an activation micro-switch in the core of mGlu4, in agreement with previously published experimental information where mutations of this residue reduce MPEP activity, as well as that of other mGlu4 PAMs^32^. It is possible this conserved residue functions in the same way in all mGluRs, including mGlu5 and mGlu8 where potency of several mGlu5 PAMs is reduced upon its mutation^58, 62^ (sometimes turning a PAM into a NAM)^62^ or generating constitutive receptor activity^60^. This microswitch ability appears to operate by maintaining mGlu4 in an inactive state through H-bonding with opposite residues on TM6/7, and upon breaking these inter-helical interactions through H-bonding with a PAM, Y3.44 then applies translational/rotational forces on TM3. This encourages the receptor to adopt a more active-like state.

The conformational changes observed here in ICL1 and ICL2 of mGlu4 can be put in context by comparison with experimental FRET^35^ and cross-linking^61^ studies of mGlu1 and mGlu8, respectively. Although not the same receptor, it might be expected that mGluR activation follows similar lines across family members (as observed in Class A GPCRs)^75, 80, 83^. As such, FRET measurements of the ICL2/C-terminal distance were found to change during mGlu1 activation, suggesting ICL2 conformational change occurs during mGluR activation^35^. Likewise, when ICL1 and ICL2 are cross-linked by cysteine mutations, it suppresses mGlu8 activation by preventing loop conformational changes^61^. This further supports the observations made here in mGlu4 where the distance between ICL1 and ICL2 widens as the receptor undergoes positive allosteric modulation, with ICL2 adopting an outward position and ICL1 partially disordering.

In native conditions, mGluRs bind glutamate in their extracellular orthosteric site but whether they also bind an endogenous allosteric modulator in their TM domains is unknown. There is notable sequence variation amongst mGluRs in their TM allosteric sites, as seen here with mGlu4 and mGlu5, and it is possible that these differences are not accidental. Instead, they could provide an additional way of controlling receptor function *in vivo* through discriminatory binding of other elements, in the same way as their extracellular domains are allosterically regulated by chloride ions^84^. Although speculative, we find it interesting that the apo state of mGlu5 is able to bind fatty acid chains of lipid molecules in its allosteric site, in a similar fashion to the Class A GPCR sphingosine 1-phosphate receptor 1^43^. However, in this case the pathway of lipid entry is between TM5 and TM6, primarily mediated by a flexible tryptophan residue. Indeed, this may provide a form of allosteric modulation, by contributing to the stabilization of the inactive state. It is also interesting to note that lipid binding does not occur when MPEP is bound, suggesting the binding of allosteric modulators and lipids are not compatible. Although more research concerning lipid binding is required, the MD simulations performed here provide a glimpse of what might be endogenous allosteric regulation in the mGlu5 TM domain.

## Conclusions

By comparing the functionality of a single ligand, MPEP, in two different receptors, mGlu4 and mGlu5, we focus on receptor recognition modes that discriminate PAM and NAM functionality, whilst avoiding ligand structure-activity noise. Through homology modelling and unbiased long-timescale MD simulations, two different MPEP binding modes are identified in mGlu4 and mGlu5 (SI Fig. 12), containing two different protein-ligand H-bonds via conserved residues on either TM3 (Y3.44) or TM7 (S7.39), respectively. In particular, a key residue determinant appears to be C2.49 (mGlu4) or G2.49 (mGlu5), which changes the size and nature of the allosteric pocket. In mGlu4, MPEP causes receptor activation by changing the conformation of TM3, TM4 and TM5, destabilizing the ionic-lock, and separating ICL1, ICL2 and ICL3. On the contrary, mGlu5 remains stable and inactive with bound MPEP. These findings may provide a mechanistic explanation regarding mGluR activation in general.

## Electronic supplementary material

Supplementary Information

## References

[1] Pin JP, Acher F (2002). The metabotropic glutamate receptors: structure, activation mechanism and pharmacology. Current drug targets. CNS and neurological disorders.

[2] Pin JP, Galvez T, Prezeau L (2003). Evolution, structure, and activation mechanism of family 3/C G-protein-coupled receptors. Pharmacology & therapeutics.

[3] Kniazeff J, Prezeau L, Rondard P, Pin JP, Goudet C (2011). Dimers and beyond: The functional puzzles of class C GPCRs. Pharmacology & therapeutics.

[4] Binet V (2007). Common structural requirements for heptahelical domain function in class A and class C G protein-coupled receptors. The Journal of biological chemistry.

[5] Goudet C (2004). Heptahelical domain of metabotropic glutamate receptor 5 behaves like rhodopsin-like receptors. Proceedings of the National Academy of Sciences of the United States of America.

[6] Rondard P, Pin JP (2015). Dynamics and modulation of metabotropic glutamate receptors. Current opinion in pharmacology.

[7] Featherstone DE (2010). Intercellular glutamate signaling in the nervous system and beyond. ACS chemical neuroscience.

[8] Niswender CM, Conn PJ (2010). Metabotropic glutamate receptors: physiology, pharmacology, and disease. Annual review of pharmacology and toxicology.

[9] Nicoletti F (2011). Metabotropic glutamate receptors: from the workbench to the bedside. Neuropharmacology.

[10] Flor PJ, Acher FC (2012). Orthosteric versus allosteric GPCR activation: the great challenge of group-III mGluRs. Biochemical pharmacology.

[11] Burford NT, Watson J, Bertekap R, Alt A (2011). Strategies for the identification of allosteric modulators of G-protein-coupled receptors. Biochemical pharmacology.

[12] Conn PJ, Christopoulos A, Lindsley CW (2009). Allosteric modulators of GPCRs: a novel approach for the treatment of CNS disorders. Nature reviews. Drug discovery.

[13] Pin JP, Duvoisin R (1995). The metabotropic glutamate receptors: structure and functions. Neuropharmacology.

[14] Raber J, Duvoisin RM (2015). Novel metabotropic glutamate receptor 4 and glutamate receptor 8 therapeutics for the treatment of anxiety. Expert opinion on investigational drugs.

[15] Swanson CJ (2005). Metabotropic glutamate receptors as novel targets for anxiety and stress disorders. Nature reviews. Drug discovery.

[16] Nickols HH (2016). VU0477573: Partial Negative Allosteric Modulator of the Subtype 5 Metabotropic Glutamate Receptor with *In Vivo* Efficacy. The Journal of pharmacology and experimental therapeutics.

[17] Zhou Q (2013). Effect of metabotropic glutamate 5 receptor antagonists on morphine efficacy and tolerance in rats with neuropathic pain. European journal of pharmacology.

[18] Waung MW, Akerman S, Wakefield M, Keywood C, Goadsby PJ (2016). Metabotropic glutamate receptor 5: a target for migraine therapy. Annals of clinical and translational neurology.

[19] Vincent K (2016). Intracellular mGluR5 plays a critical role in neuropathic pain. Nature communications.

[20] Vilar B (2013). Alleviating pain hypersensitivity through activation of type 4 metabotropic glutamate receptor. The Journal of neuroscience: the official journal of the Society for Neuroscience.

[21] Acher F, Goudet C (2015). Therapeutic potential of group III metabotropic glutamate receptor ligands in pain. Current opinion in pharmacology.

[22] Dickerson JW, Conn PJ (2012). Therapeutic potential of targeting metabotropic glutamate receptors for Parkinson’s disease. Neurodegenerative disease management.

[23] Nickols HH, Conn PJ (2014). Development of allosteric modulators of GPCRs for treatment of CNS disorders. Neurobiology of disease.

[24] Mathiesen JM, Svendsen N, Brauner-Osborne H, Thomsen C, Ramirez MT (2003). Positive allosteric modulation of the human metabotropic glutamate receptor 4 (hmGluR4) by SIB-1893 and MPEP. British journal of pharmacology.

[25] O’Brien JA (2003). A family of highly selective allosteric modulators of the metabotropic glutamate receptor subtype 5. Molecular pharmacology.

[26] O’Brien JA (2004). A novel selective allosteric modulator potentiates the activity of native metabotropic glutamate receptor subtype 5 in rat forebrain. The Journal of pharmacology and experimental therapeutics.

[27] Sheffler DJ, Gregory KJ, Rook JM, Conn PJ (2011). Allosteric modulation of metabotropic glutamate receptors. Adv Pharmacol.

[28] Christopher JA (2015). Fragment and Structure-Based Drug Discovery for a Class C GPCR: Discovery of the mGlu5 Negative Allosteric Modulator HTL14242 (3-Chloro-5-[6-(5-fluoropyridin-2-yl)pyrimidin-4-yl]benzonitrile. Journal of medicinal chemistry.

[29] Dore AS (2014). Structure of class C GPCR metabotropic glutamate receptor 5 transmembrane domain. Nature.

[30] Wu H (2014). Structure of a class C GPCR metabotropic glutamate receptor 1 bound to an allosteric modulator. Science.

[31] Dalton, J. A. *et al*. Shining Light On An mGlu5 Photoswitchable NAM: A Theoretical Perspective. *Current neuropharmacology* (2015). 10.2174/1570159X13666150407231417 PMC4983757 26391742

[32] Rovira X (2015). Overlapping binding sites drive allosteric agonism and positive cooperativity in type 4 metabotropic glutamate receptors. FASEB journal: official publication of the Federation of American Societies for Experimental Biology.

[33] Bennett KA, Dore AS, Christopher JA, Weiss DR, Marshall FH (2015). Structures of mGluRs shed light on the challenges of drug development of allosteric modulators. Current opinion in pharmacology.

[34] Xue L (2015). Major ligand-induced rearrangement of the heptahelical domain interface in a GPCR dimer. Nature chemical biology.

[35] Hlavackova V (2012). Sequential inter- and intrasubunit rearrangements during activation of dimeric metabotropic glutamate receptor 1. Science signaling.

[36] Binet V (2004). The heptahelical domain of GABA(B2) is activated directly by CGP7930, a positive allosteric modulator of the GABA(B) receptor. The Journal of biological chemistry.

[37] Ray K (2005). Calindol, a positive allosteric modulator of the human Ca(2+) receptor, activates an extracellular ligand-binding domain-deleted rhodopsin-like seven-transmembrane structure in the absence of Ca(2+). The Journal of biological chemistry.

[38] Latorraca, N. R., Venkatakrishnan, A. J. & Dror, R. O. GPCR Dynamics: Structures in Motion. *Chemical reviews*, doi:10.1021/acs.chemrev.6b00177 (2016). 10.1021/acs.chemrev.6b00177 27622975

[39] Hertig S, Latorraca NR, Dror RO (2016). Revealing Atomic-Level Mechanisms of Protein Allostery with Molecular Dynamics Simulations. PLoS computational biology.

[40] Miao Y, McCammon JA (2016). G-protein coupled receptors: advances in simulation and drug discovery. Current opinion in structural biology.

[41] Perez-Aguilar JM, Shan J, LeVine MV, Khelashvili G, Weinstein H (2014). A functional selectivity mechanism at the serotonin-2A GPCR involves ligand-dependent conformations of intracellular loop 2. Journal of the American Chemical Society.

[42] Ozgur C, Doruker P, Akten ED (2016). Investigation of allosteric coupling in human beta2-adrenergic receptor in the presence of intracellular loop 3. BMC structural biology.

[43] Stanley N, Pardo L, Fabritiis GD (2016). The pathway of ligand entry from the membrane bilayer to a lipid G protein-coupled receptor. Scientific reports.

[44] Nygaard R (2013). The dynamic process of beta(2)-adrenergic receptor activation. Cell.

[45] Huang W (2015). Structural insights into micro-opioid receptor activation. Nature.

[46] Ye L, Van Eps N, Zimmer M, Ernst OP, Prosser RS (2016). Activation of the A2A adenosine G-protein-coupled receptor by conformational selection. Nature.

[47] Casiraghi M (2016). Functional Modulation of a G Protein-Coupled Receptor Conformational Landscape in a Lipid Bilayer. Journal of the American Chemical Society.

[48] Kufareva I, Katritch V, Stevens RC, Abagyan R (2014). Advances in GPCR modeling evaluated by the GPCR Dock 2013 assessment: meeting new challenges. Structure.

[49] Huang J, MacKerell AD (2013). CHARMM36 all-atom additive protein force field: validation based on comparison to NMR data. Journal of computational chemistry.

[50] Piggot TJ, Pineiro A, Khalid S (2012). Molecular Dynamics Simulations of Phosphatidylcholine Membranes: A Comparative Force Field Study. J Chem Theory Comput.

[51] Pluhackova K (2016). A Critical Comparison of Biomembrane Force Fields: Structure and Dynamics of Model DMPC, POPC, and POPE Bilayers. The journal of physical chemistry. B.

[52] Vanommeslaeghe K (2010). CHARMM general force field: A force field for drug-like molecules compatible with the CHARMM all-atom additive biological force fields. Journal of computational chemistry.

[53] Vanommeslaeghe K, MacKerell AD (2012). Automation of the CHARMM General Force Field (CGenFF) I: bond perception and atom typing. Journal of chemical information and modeling.

[54] Vanommeslaeghe K, Raman EP, MacKerell AD (2012). Automation of the CHARMM General Force Field (CGenFF) II: assignment of bonded parameters and partial atomic charges. Journal of chemical information and modeling.

[55] Mayne CG, Saam J, Schulten K, Tajkhorshid E, Gumbart JC (2013). Rapid parameterization of small molecules using the Force Field Toolkit. Journal of computational chemistry.

[56] Lans I, Dalton JA, Giraldo J (2015). Selective Protonation of Acidic Residues Triggers Opsin Activation. The journal of physical chemistry. B.

[57] Conn PJ, Lindsley CW, Meiler J, Niswender CM (2014). Opportunities and challenges in the discovery of allosteric modulators of GPCRs for treating CNS disorders. Nature reviews. Drug discovery.

[58] Gregory KJ (2014). Identification of specific ligand-receptor interactions that govern binding and cooperativity of diverse modulators to a common metabotropic glutamate receptor 5 allosteric site. ACS chemical neuroscience.

[59] Pin JP, Gomeza J, Joly C, Bockaert J (1995). The metabotropic glutamate receptors: their second intracellular loop plays a critical role in the G-protein coupling specificity. Biochemical Society transactions.

[60] Yanagawa M, Yamashita T, Shichida Y (2009). Activation switch in the transmembrane domain of metabotropic glutamate receptor. Molecular pharmacology.

[61] Yamashita T, Terakita A, Kai T, Shichida Y (2008). Conformational change of the transmembrane helices II and IV of metabotropic glutamate receptor involved in G protein activation. Journal of neurochemistry.

[62] Gregory KJ (2013). Probing the metabotropic glutamate receptor 5 (mGlu(5)) positive allosteric modulator (PAM) binding pocket: discovery of point mutations that engender a “molecular switch” in PAM pharmacology. Molecular pharmacology.

[63] Chivian D, Baker D (2006). Homology modeling using parametric alignment ensemble generation with consensus and energy-based model selection. Nucleic acids research.

[64] Kim DE, Chivian D, Baker D (2004). Protein structure prediction and analysis using the Robetta server. Nucleic acids research.

[65] Pei J, Kim BH, Grishin NV (2008). PROMALS3D: a tool for multiple protein sequence and structure alignments. Nucleic acids research.

[66] Schrödinger Release 2014-2: Maestro, version 9.8, Schrödinger, LLC, New York, NY (2014).

[67] Morris GM (2009). AutoDock4 and AutoDockTools4: Automated docking with selective receptor flexibility. Journal of computational chemistry.

[68] Pettersen EF (2004). UCSF chimera - A visualization system for exploratory research and analysis. Journal of computational chemistry.

[69] AMBER 14, University of California, San Francisco (2014).

[70] Jo S, Kim T, Iyer VG, Im W (2008). CHARMM-GUI: a web-based graphical user interface for CHARMM. Journal of computational chemistry.

[71] Lomize MA, Lomize AL, Pogozheva ID, Mosberg HI (2006). OPM: orientations of proteins in membranes database. Bioinformatics.

[72] Harvey MJ, Giupponi G, De Fabritiis G (2009). ACEMD: Accelerating Biomolecular Dynamics in the Microsecond Time Scale. J Chem Theory Comput.

[73] Humphrey W, Dalke A, Schulten K (1996). VMD: visual molecular dynamics. Journal of molecular graphics.

[74] Dalton JAR, Michalopoulos I, Westhead DR (2003). Calculation of helix packing angles in protein structures. Bioinformatics.

[75] Dalton JA, Lans I, Giraldo J (2015). Quantifying conformational changes in GPCRs: glimpse of a common functional mechanism. BMC bioinformatics.

[76] Kabsch W, Sander C (1983). Dictionary of protein secondary structure: pattern recognition of hydrogen-bonded and geometrical features. Biopolymers.

[77] Simons KT, Kooperberg C, Huang E, Baker D (1997). Assembly of protein tertiary structures from fragments with similar local sequences using simulated annealing and Bayesian scoring functions. Journal of molecular biology.

[78] Nowroozi, A. & Shahlaei, M. A coupling of homology modeling with multiple molecular dynamics simulation for identifying representative conformation of GPCR structures: a case study on human bombesin receptor subtype-3. *Journal of biomolecular structure & dynamics*, 1–23, doi:10.1080/07391102.2016.1140593 (2016). 10.1080/07391102.2016.1140593 26922838

[79] Harpsoe K (2015). Selective Negative Allosteric Modulation Of Metabotropic Glutamate Receptors - A Structural Perspective of Ligands and Mutants. Scientific reports.

[80] Tehan BG, Bortolato A, Blaney FE, Weir MP, Mason JS (2014). Unifying family A GPCR theories of activation. Pharmacology & therapeutics.

[81] Rasmussen SG (2011). Crystal structure of the beta2 adrenergic receptor-Gs protein complex. Nature.

[82] Kruse AC (2013). Activation and allosteric modulation of a muscarinic acetylcholine receptor. Nature.

[83] Lans I, Dalton JA, Giraldo J (2015). Helix 3 acts as a conformational hinge in Class A GPCR activation: An analysis of interhelical interaction energies in crystal structures. Journal of structural biology.

[84] Tora AS (2015). Allosteric modulation of metabotropic glutamate receptors by chloride ions. FASEB journal: official publication of the Federation of American Societies for Experimental Biology.

