# Supplementary material for: Analysis of positive and negative allosteric modulation in metabotropic glutamate receptors 4 and 5 with a dual ligand
Source: Sci Rep. 2017 Jul 10;7:4944. doi: 10.1038/s41598-017-05095-5 (PMC5504000; doi:10.1038/s41598-017-05095-5)
Supplement: Supplementary file 1 — Supplementary Information [file 41598_2017_5095_MOESM1_ESM.pdf]

Analysis of positive and negative allosteric modulation in metabotropic glutamate receptors 4 and 5 with a dual ligand

James A.R. Dalton[1], Jean-Philippe Pin[2,3], Jesús Giraldo[1,*]

[1] Laboratory of Molecular Neuropharmacology and Bioinformatics, Institut de Neurociències and Unitat de Bioestadística, Universitat Autònoma de Barcelona, 08193 Bellaterra, Spain; Network Biomedical Research Centre on Mental Health (CIBERSAM), Spain.
[2] Institute of Functional Genomics, Université de Montpellier, Unité Mixte de Recherche 5302 CNRS, Montpellier, France.
[3] Unité de recherche U1191, INSERM, Montpellier, France.

Supplementary Information

```
mglu4     1 IPIIKLEWGSPWAVLPLFLAVVGIAATLFVVITFVRYNDTPIVKASGRELSYVLLAGIFL
mglu5     1 IPVQYLRWGDPEPIAAVVFACLGLLATLFVTVVFIIYRDTPVVKSSSRELCYIILAGICL

mglu4    61 CYATTFLMIAEPDLGTCSLRRIFLGLGMSISYAALLTKTNRIYRIFEQGKRSV--SAPRF
mglu5    61 GYLCTFCLIAKPKQIYCYLQRIGIGLSPAMSYSALVTKTNRIARILAGSKKKICTKKPRF

mglu4   119 ISPASQLAITFSLISLQLLGICVWFVVDPSHSVVDFQDQRTLDPRFARGVLKCDISDLSL
mglu5   121 MSACAQLVIAFILICIQLGIIVALFIMEPPDIMHDYPSIR-------EVYLICNTTNLGV

mglu4   179 ICLLGYSMLLMVTCTVYAIKTRGVPETFNEAKPIGFTMYTTCIVWLAFIPIFFGTSQSAD
mglu5   174 VTPLGYNGLLILSCTFYAFKTRNVPANFNEAKYIAFTMYTTCIIWLAFVPIYFGSNY---

mglu4   239 KLYIQTTTLTVSVSLSASVSLGMLYMPKVYIILFHPEQN
mglu5   231 ----KIITMCFSVSLSATVALGCMFVPKVYIILAKPERN
```

SI Figure 1. Sequence alignment between transmembrane domains of human mGlu4 and mGlu5.

SI Table 1. Predicted docking scores (from Autodock4.2) of MPEP in mGlu4 homology model and mGlu5 crystal structure (PDB id: 4OO9)

	mGlu5	mGlu4
**Predicted docking score** (estimated free energy of binding, kcal/mol)	-7.32	-6.96

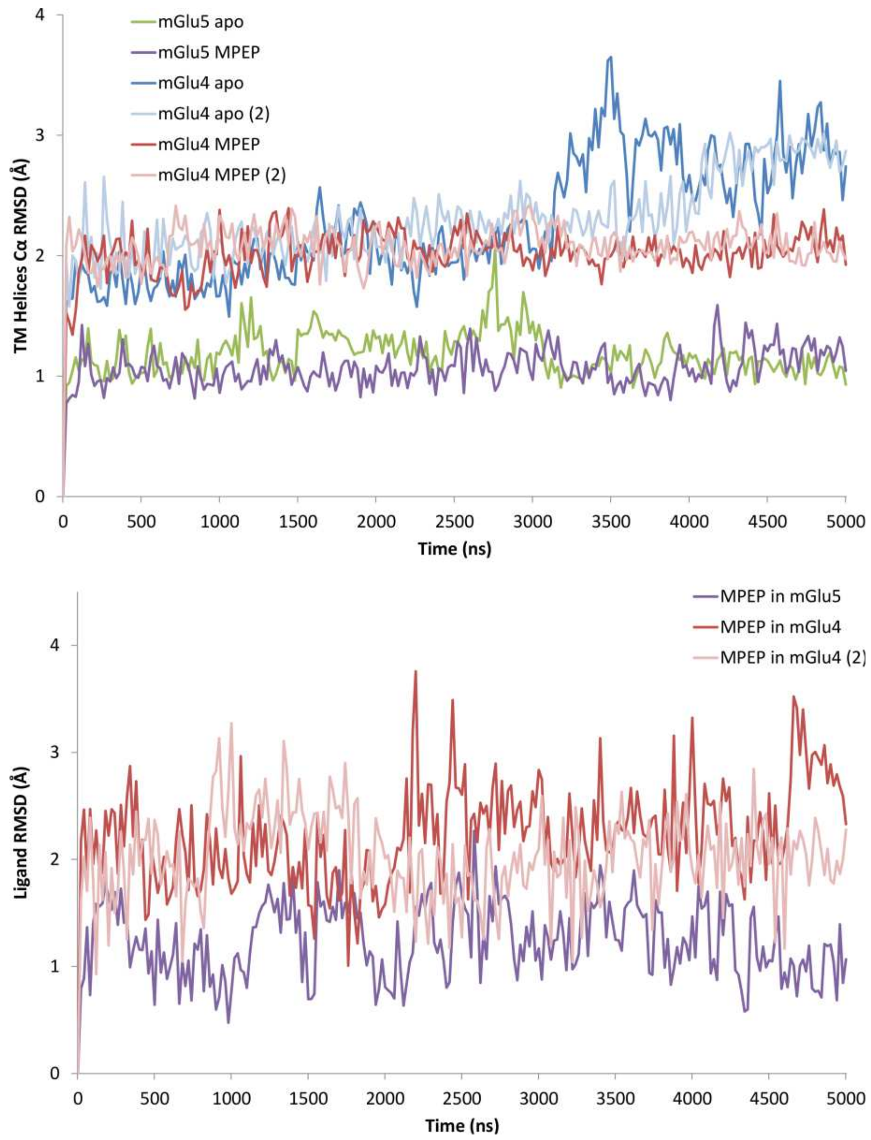

SI Figure 2. Top: RMSD of mGlu4 and mGlu5 transmembrane domains (Cα atoms of TM helices) in apo and MPEP-bound states in respective 5-microsecond MD simulations (calculated with respect to starting protein structure). MD simulations of mGlu4 are duplicated. Bottom: RMSD of allosteric modulator MPEP, bound in mGlu4 and mGlu5 (calculated with respect to original docking).

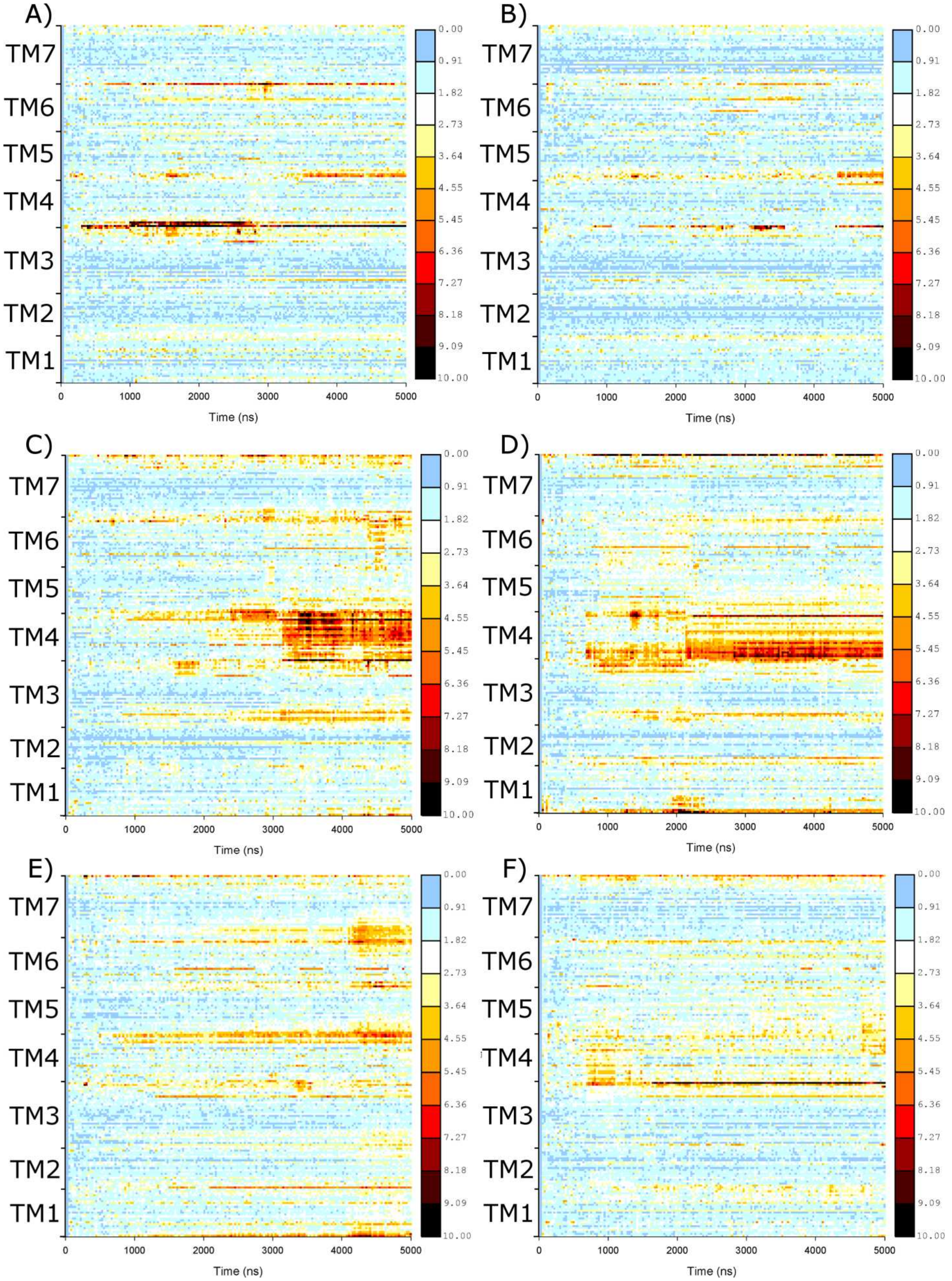

SI Figure 3. Conformational fluctuation heatmaps (RMSF of TM helix Cα atoms, normalized magnitude from 1 to 10) of: A) apo mGlu5, B) MPEP-bound mGlu5, C) and E) apo mGlu4, D) and F) MPEP-bound mGlu4, over respective 5 µs MD simulations (mGlu4 MD simulations performed in duplicate; fluctuations calculated every 20 ns with respect to previous conformation).

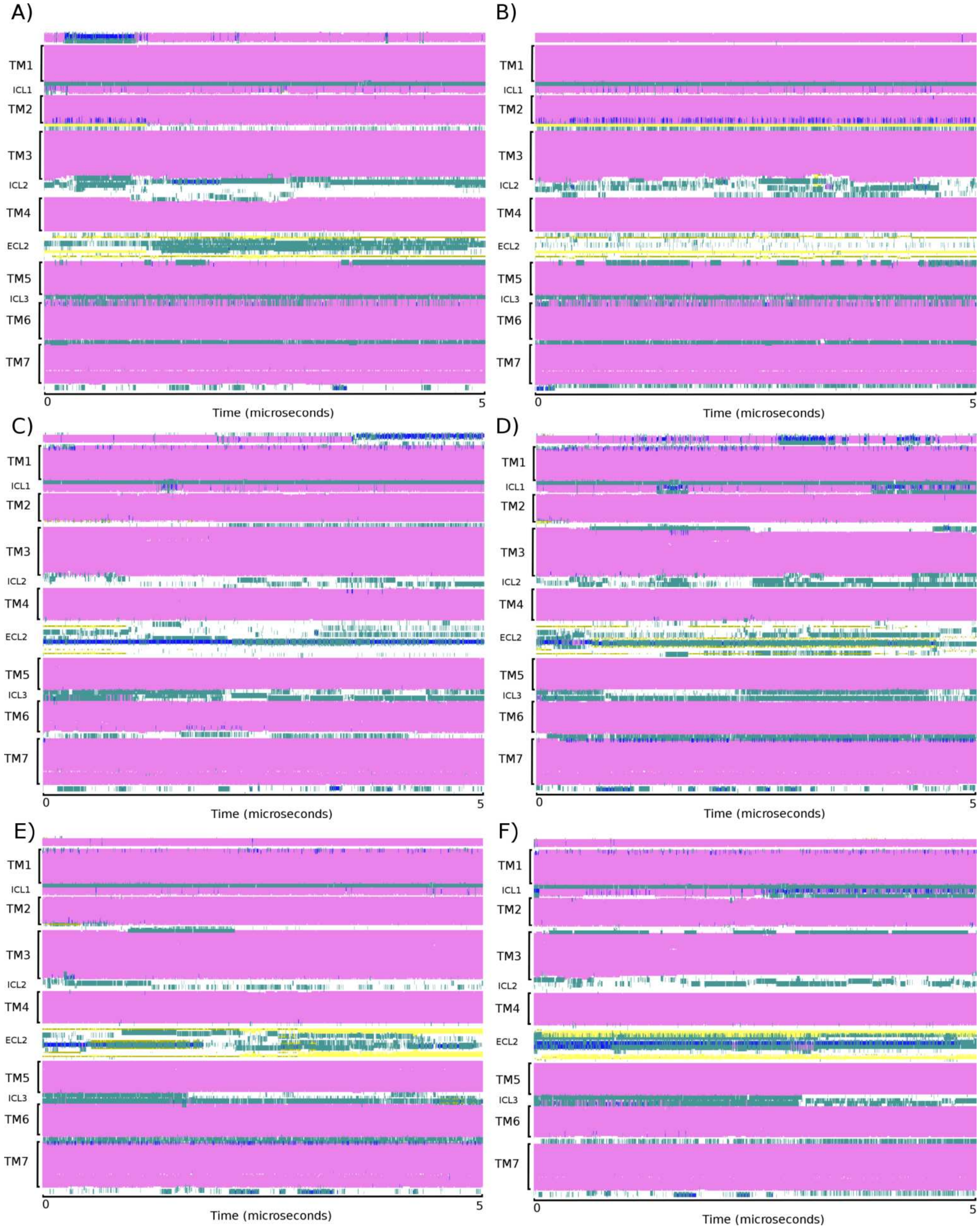

SI Figure 4. A secondary structure analysis of (a) apo mGlu5, (b) mGlu5 bound to MPEP, (c) and (e) apo mGlu4, (d) and (f) mGlu4 bound to MPEP, over respective MD simulations of 5 microseconds each (MD simulations of mGlu4 are duplicated). Colours: pink: α-helix, blue: 310-helix, yellow: beta-strand, teal: turn, white: coil.

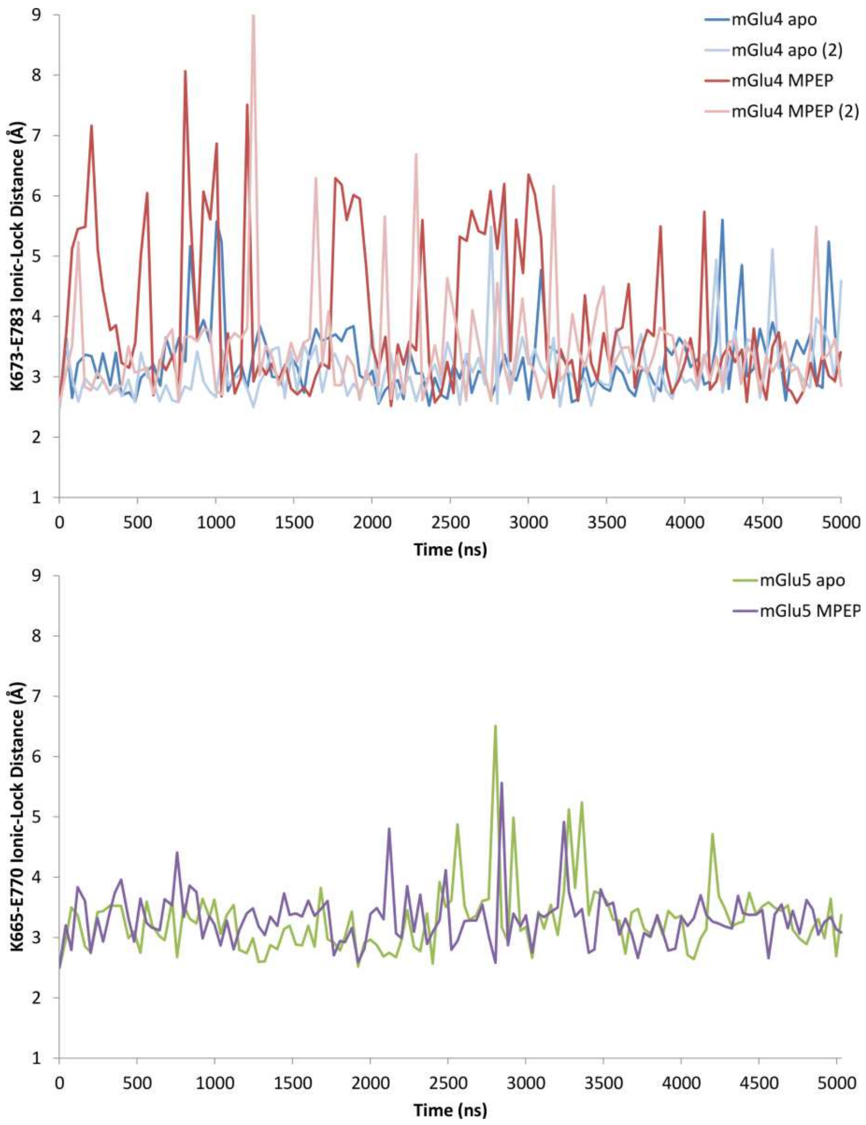

SI Figure 5. Top: ionic-lock distance in mGlu4 (N---O, K673-E783) and bottom: ionic-lock distance in mGlu5 (N---O, K665-E770) in respective 5 microsecond MD simulations, with and without bound allosteric modulator, MPEP. MD simulations of mGlu4 are duplicated.

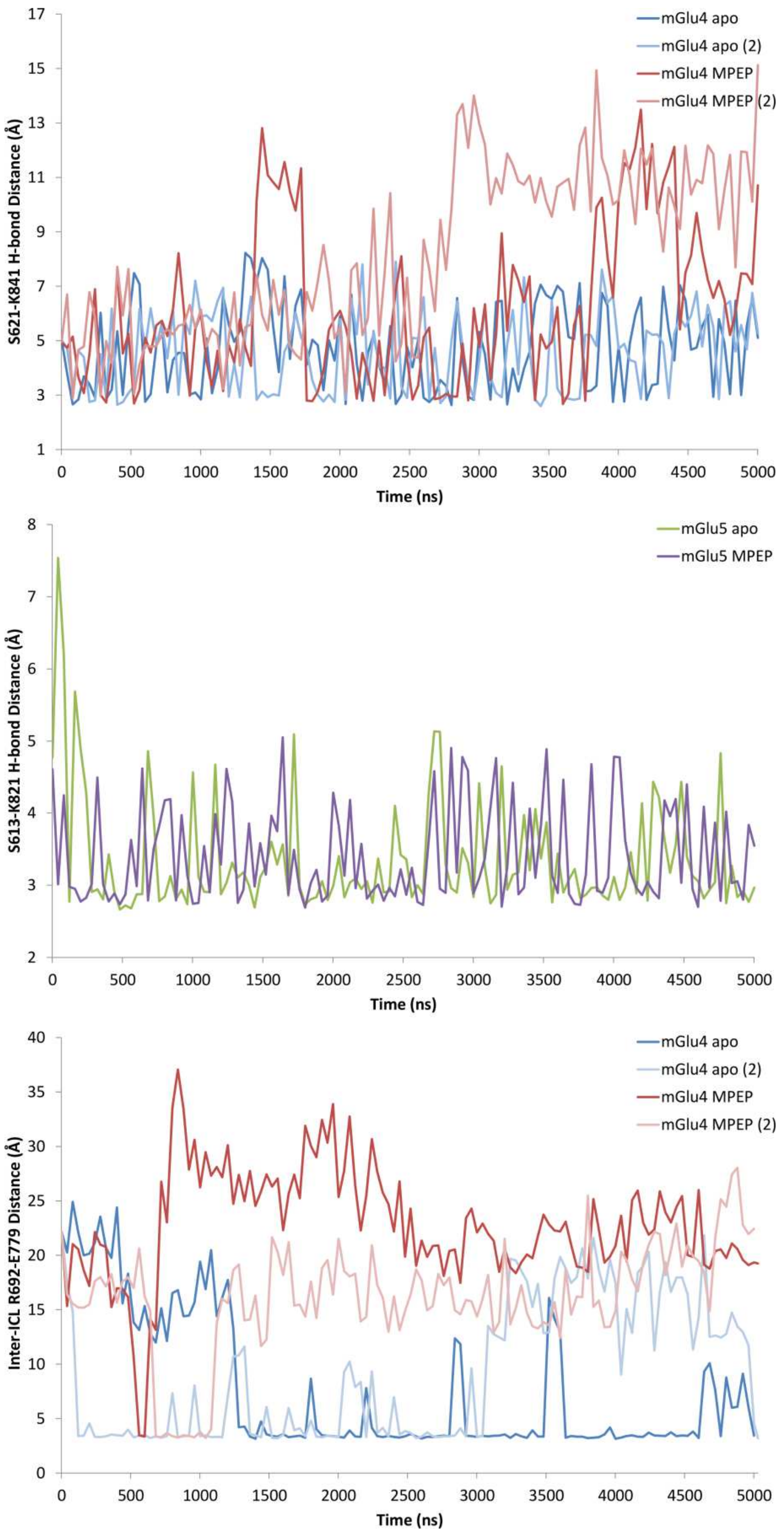

SI Figure 6. Additional intramolecular locking interactions observed in MD simulations of mGlu4 and mGlu5 performed with and without bound allosteric modulator, MPEP. Top**:** S621-K841 (ICL1-TM7) H-bond distance in mGlu4 (O---N); middle: S613-K821 (ICL1-TM7) H-bond distance in mGlu5 (O---N); bottom: inter-loop R692-E779 salt-bridge (ICL2-ICL3) in mGlu4. MD simulations of mGlu4 are duplicated (5 microseconds each).

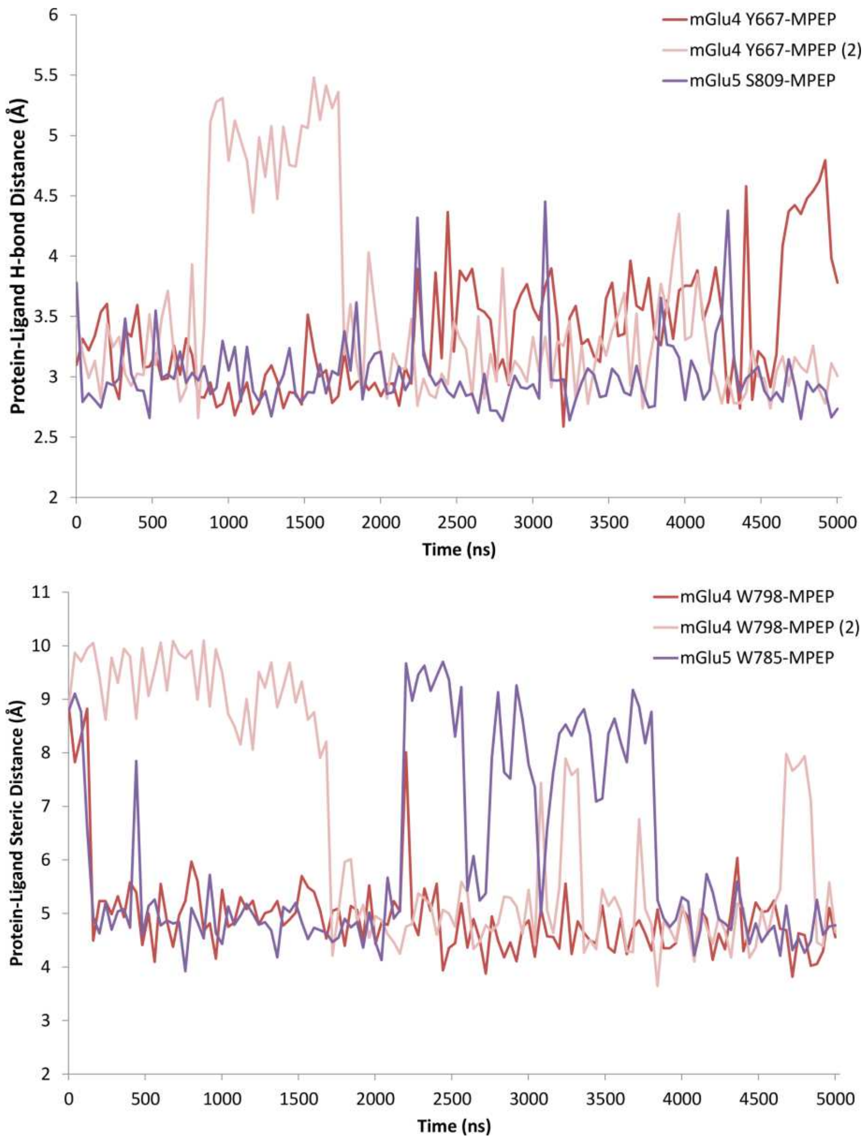

SI Figure 7. Assessment of observed protein-ligand interactions in MD simulations of mGlu4 and mGlu5 with bound allosteric modulator, MPEP, over 5 microseconds. Top**:** protein-ligand H-bond distances in mGlu4 (Y667-MPEP, O---N) and mGlu5 (S809-MPEP, O---N). Bottom: protein-ligand contact distances between MPEP and W798/W785$^{6.50}$ in MD simulations of mGlu4 and mGlu5, respectively (calculated between Trp sidechain N atom and MPEP centre of mass). MD simulations of mGlu4 are duplicated.

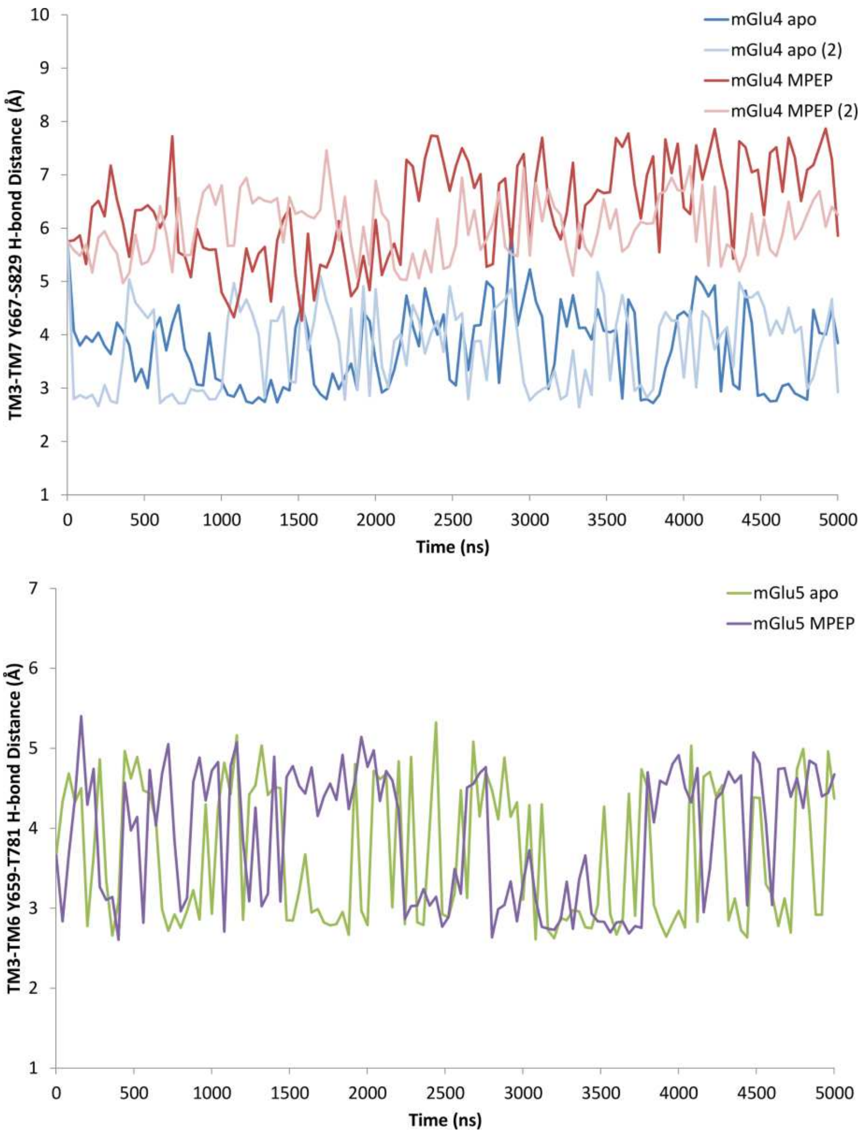

SI Figure 8. Assessment of observed H-bonds (or breakage) in TM domain cores of mGlu4 and mGlu5. MD simulations are performed with and without bound allosteric modulator, MPEP, over 5 microseconds each. Top**:** Y667-S829 (TM3-TM7) H-bond distance in mGlu4 (O---O); Bottom: Y659-T781 (TM3-TM6) H-bond distance in mGlu5 (O---O). MD simulations of mGlu4 are duplicated.

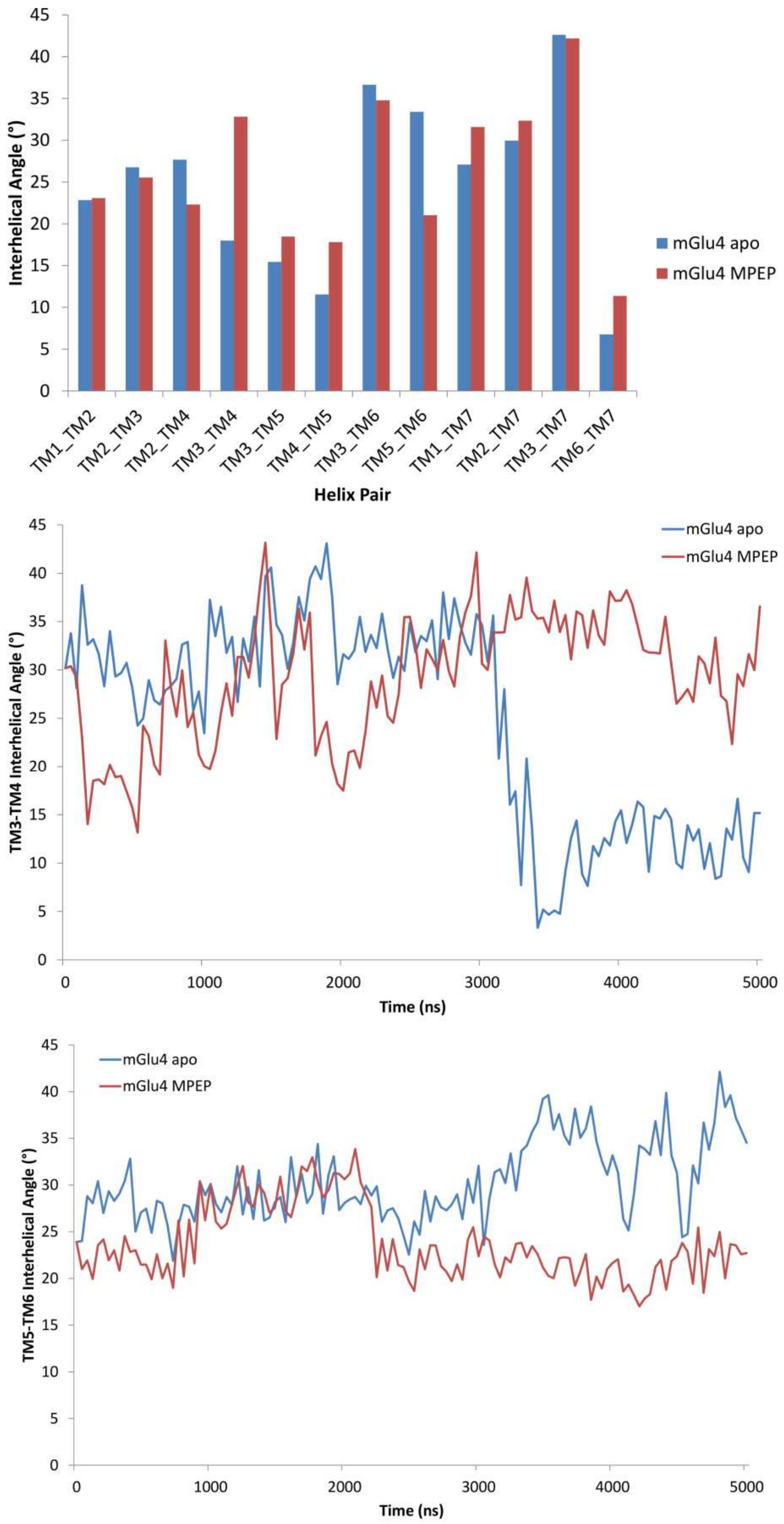

SI Figure 9. Comparison of inter-helical angles for quantification of TM domain conformational changes in mGlu4, with and without bound allosteric modulator, MPEP. (A) Twelve inter-helical angles (between helix pairs containing inter-helical vdW contacts) observed in average apo- and MPEP-bound mGlu4 states, retrieved from respective 5-microsecond MD simulations (average state calculated from second half of simulation). (B and C) Variation of TM3-TM4 (B) and TM5-TM6 (C) inter-helical angles observed during MD simulations of mGlu4 with and without bound MPEP.

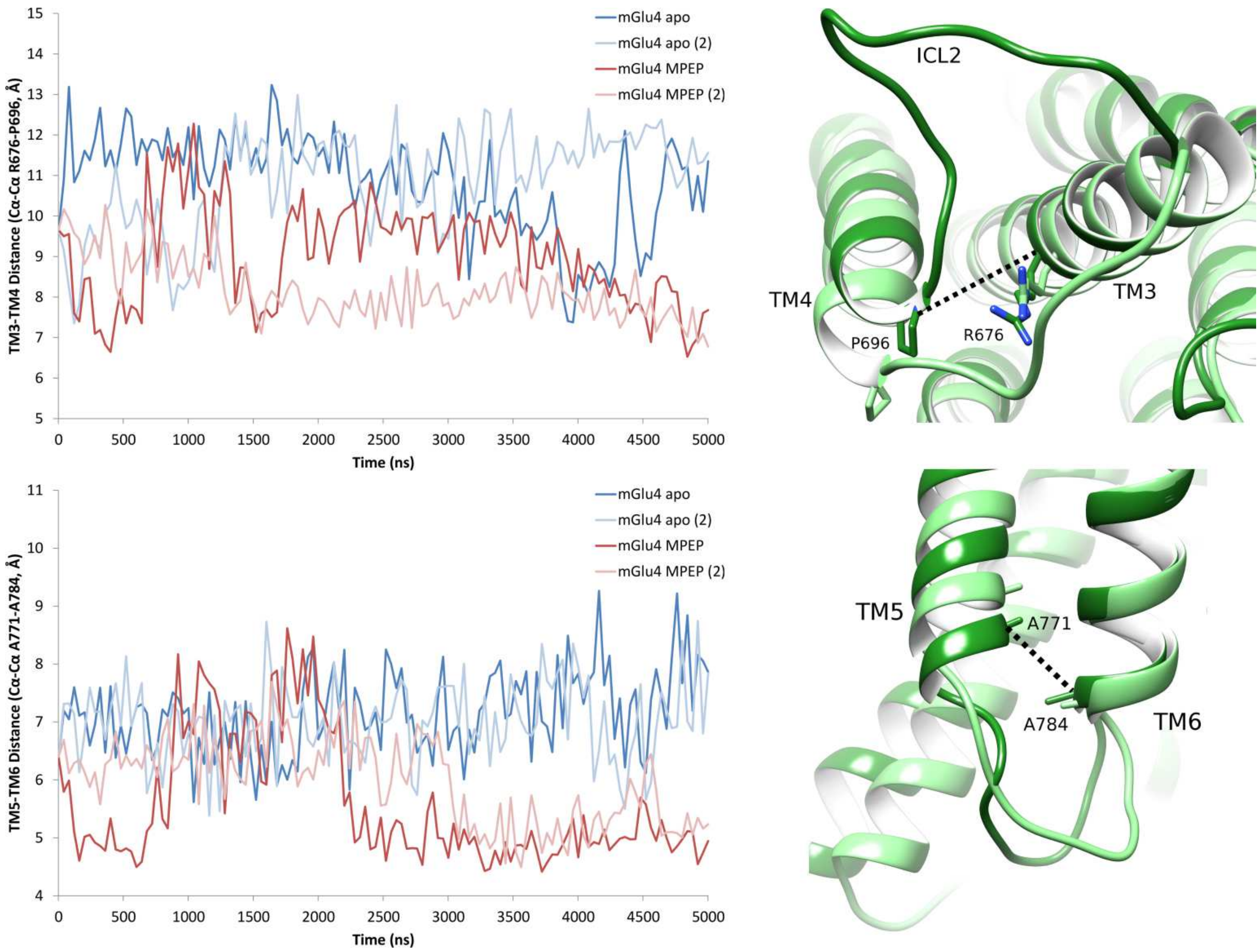

SI Figure 10. Comparison of inter-helical distances for quantification of TM domain conformational changes in mGlu4, with and without bound allosteric modulator, MPEP. Variation of TM3-TM4 (top) and TM5-TM6 (bottom) distances observed during respective 5-microsecond MD simulations of mGlu4, with and without bound MPEP. Inter-helical distances are measured at intracellular side between Cα atoms of residue pairs: R676 and P696 (TM3-TM4), A771 and A784 (TM5-TM6). Corresponding structural images (right) show relevant inter-helical distances between average states of mGlu4, either MPEP-bound (dark green) or apo (light green), obtained from their respective 5-microsecond MD simulations.

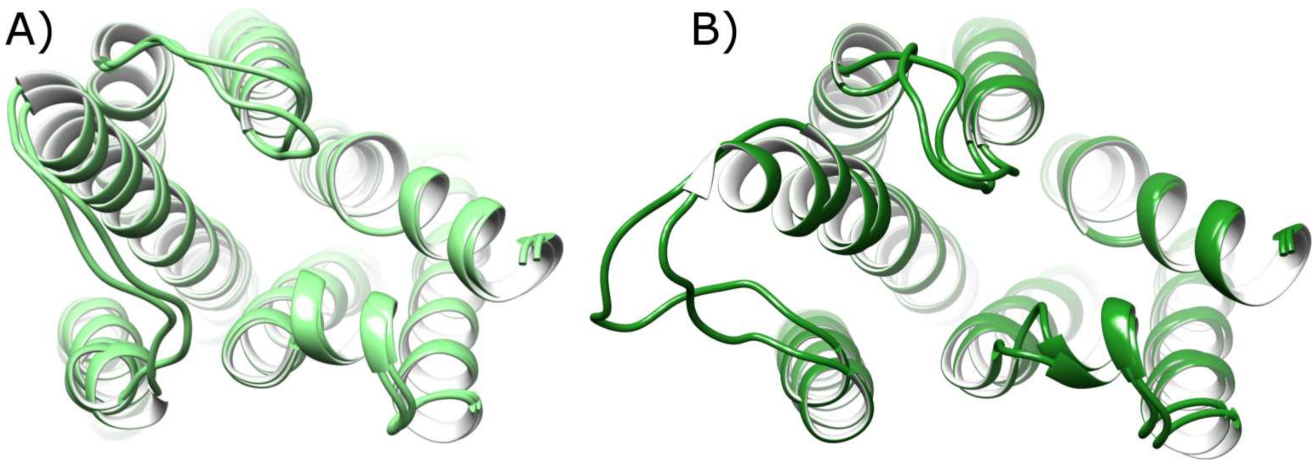

SI Figure 11. Comparison between intracellular conformations of duplicated average states of mGlu4 in: A) apo state (light green) or B) MPEP-bound (dark green), obtained from respective 5-µs MD simulations (average conformations calculated from 2.5-5 µs). Original and duplicated conformations are superimposed.

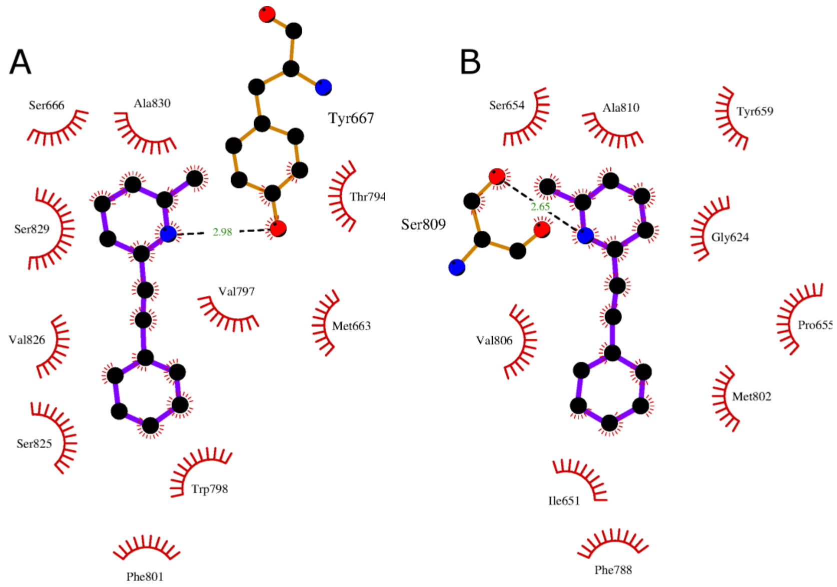

SI Figure 12. A 2-D comparison of average observed MPEP binding in MD simulations of (A) mGlu4 and (B) mGlu5.
